# Targeting selenoprotein H in the nucleolus suppresses tumors and metastases by Isovalerylspiramycin I

**DOI:** 10.1186/s13046-022-02350-0

**Published:** 2022-04-06

**Authors:** Jing Cui, Jingcheng Zhou, Weiqing He, Juan Ye, Timothy Westlake, Rogelio Medina, Herui Wang, Bhushan L. Thakur, Juanjuan Liu, Mingyu Xia, Zhonggui He, Fred E. Indig, Aiguo Li, Yan Li, Robert J. Weil, Mirit I. Aladjem, Laiping Zhong, Mark R. Gilbert, Zhengping Zhuang

**Affiliations:** 1 Neuro-Oncology Branch, National Cancer Institute, Center for Cancer Research, National Institutes of Health, Building 35 Room 2B203 35 Convent Dr., Bethesda, MD 20892 USA; 2 CAMS Key Laboratory of Synthetic Biology for Drug Innovation, Institute of Medicinal Biotechnology, Chinese Academy of Medical Sciences & Peking Union Medical College, Peking, 100050 China; 3 Developmental Therapeutics Branch, Center for Cancer Research, National Cancer Institute, National Institutes of Health, Bethesda, MD 20892 USA; 4 School of Life Science and Biopharmaceutics, Shenyang Pharmaceutical University, 103 Wenhua Road, Shenyang, 110016 China; 5 Department of Pharmaceutics, Wuya College of Innovation, Shenyang Pharmaceutical University, 103 Wenhua Road, Shenyang, 110016 China; 6 Confocal Imaging Facility, National Institute On Aging, National Institutes of Health, Baltimore, MD 21224 USA; 7 Basic Neurosciences Program, National Institute of Neurological Disorders and Stroke, National Institutes of Health, Bethesda, MD 20892 USA; 8 College of Stomatology, Ninth People’s Hospital, Shanghai Jiao Tong University School of Medicine, Shanghai, 200011 China

**Keywords:** Carrimycin, Isovalerylspiramycin I, Malignant tumors, Metastasis, Selenoprotein H, Reactive oxygen species, Nucleolar stress, Ribosomal RNA biogenesis

## Abstract

**Background:**

Compared to normal cells, cancer cells exhibit a higher level of oxidative stress, which primes key cellular and metabolic pathways and thereby increases their resilience under oxidative stress. This higher level of oxidative stress also can be exploited to kill tumor cells while leaving normal cells intact. In this study we have found that isovalerylspiramycin I (ISP I), a novel macrolide antibiotic, suppresses cancer cell growth and tumor metastases by targeting the nucleolar protein selenoprotein H (SELH), which plays critical roles in keeping redox homeostasis and genome stability in cancer cells.

**Methods:**

We developed ISP I through genetic recombination and tested the antitumor effects using primary and metastatic cancer models. The drug target was identified using the drug affinity responsive target stability (DARTS) and mass spectrum assays. The effects of ISP I were assessed for reactive oxygen species (ROS) generation, DNA damage, R-loop formation and its impact on the JNK2/TIF-IA/RNA polymerase I (POLI) transcription pathway.

**Results:**

ISP I suppresses cancer cell growth and tumor metastases by targeting SELH. Suppression of SELH induces accumulation of ROS and cancer cell-specific genomic instability. The accumulation of ROS in the nucleolus triggers nucleolar stress and blocks ribosomal RNA transcription via the JNK2/TIF-IA/POLI pathway, causing cell cycle arrest and apoptosis in cancer cells.

**Conclusions:**

We demonstrated that ISP I links cancer cell vulnerability to oxidative stress and RNA biogenesis by targeting SELH. This suggests a potential new cancer treatment paradigm, in which the primary therapeutic agent has minimal side-effects and hence may be useful for long-term cancer chemoprevention.

**Supplementary Information:**

The online version contains supplementary material available at 10.1186/s13046-022-02350-0.

## Background

Conventional anticancer regimens typically exploit cell-killing functionalities, such as irradiation, chemotherapy and, more recently, T-cell activation. This general approach can elicit off-target toxicities and other adverse side effects, such as a dysregulated immune response. An alternative approach therefore is needed whereby the mechanism of anticancer action relies less heavily on normal cell killing and its downstream consequences.

More recently, Carrimycin, a multi-component bacterial fermentation product with antibiotic activity, was safely used in completed phase III clinical trials and showed indications of anti-cancer activity [1–4]. Carrimycin has three main components: Isovalerylspiramycin (ISP) I, II, III. Previous study showed Carrimycin and/or ISP I inhibited tumor growth of oral squamous cell carcinoma and hepatocellular carcinoma both *in vitro* and *in vivo* [3, 4]. However, the target of Carrimycin remained unknown. We have identified its molecular target(s) in cancer cells and found that the active component—ISP I—specifically suppresses selenoprotein H (SELH), a member of a family of selenocysteine-containing proteins.

Selenocysteine-containing proteins are known for their antioxidant functions relating to redox homeostasis [5, 6]. Selenoproteins have been shown to alter tumorigenesis and cancer progression by protecting DNA from oxidative damage and mitigating genomic instability [5, 6]. Within the selenoprotein family, selenoprotein H (SELH) is uniquely located in the nucleolus, a nuclear compartment for ribosome biogenesis where rRNA transcription and assembly take place [7]. Ribosomes influence a variety of cellular processes, including protein synthesis, cell cycle control, genome stability, and apoptosis [8, 9]. The nucleolus acts as a “stress sensor” because of various cellular pressures, including intracellular reactive oxygen species (ROS), prompt nucleolar structural changes and functional defects [10]. Increased oxidative stress activates c-Jun N-terminal kinase (JNK2) and inhibits the RNA polymerase I (POLI) transcription initiation factor IA (TIF-IA) in the nucleolus, which in turn down-regulates ribosomal RNA (rRNA) synthesis and induces cell cycle arrest [11]. In malignant tumor cells, a high proliferation rate correlates with elevated rRNA levels and nucleolar hypertrophy [12].

Cancer cells hijack key cellular and metabolic pathways to promote dysregulated cell growth, including states that maintain intracellular redox [13, 14]. Compared to normal cells, cancer cells exhibit higher levels of oxidative stress due to their genetic and metabolic alterations [15, 16]. Chronic elevations in ROS impose selective pressure on cancer cells to adopt more efficient mechanisms of ROS detoxification, increasing their resilience under oxidative stress [17]. Located specifically in the nucleolus, SELH suppression may disrupt cancer cell redox homeostasis and inhibit ribosome biogenesis, thereby causing cell cycle arrest and apoptosis in cancer cells. To probe the potential of targeting the nucleolus in cancer, we used ISP I, an active component of Carrimycin, to regulate SELH selectively, suppressing both primary and metastatic tumors.

## Methods

### ISP I synthesis

ISP I was isolated and purified from Carrimycin, and the purity was detected by HPLC (Waters 2695 with 2998 PDA detector) on ODS column. The molecular formula was established by HRMS (Agilent 6545B) spectrometry. The NMR data were collected by Bruker Avance III (400 MHz).

### Cells

Glioblastoma cell lines (LN229, U118, T98G and A172) were derived from American Type Culture Collection (ATCC; Manassas, VA). U251 was obtained from Sigma Aldrich (St. Louis, MO). Renal cell carcinoma cell lines (ACHN and 786-O) were obtained from ATCC, UM-RC-2 were purchased from Sigma, while RCC4 was a gift from Eric Jonasch in MD Anderson. Meningioma cell lines (IOMM-Lee, JEN and CH-157) were generated in-house (University of Utah) [18]. Mouse melanoma cell line (B16) and breast cancer cell line (4T1) were purchased from ATCC. U2OS Cells were obtained from ATCC.

### Drug affinity responsive target stability (DARTS) assay

Drug affinity responsive target stability (DARTS) assay was completed to identify the target of ISP I *in vitro*. For this assay, we used the protocol published by Lomenick et al. [19]. Briefly, LN229 cells were lysed with M-PER (Pierce) and supplemented with protease and phosphatase inhibitors. After centrifugation at 14, 000 rpm for 15 min, lysates were diluted to the same final volume and protein concentration with M-PER and proposed in TNC buffer [50 mM Tris·HCl (pH 8.0), 50 mM NaCl, 10 mM CaCl_2_]. All steps were performed on ice or at 4 °C to prevent premature protein degradation. After incubation, the protein sample was incubated with ISP I (40 μM) or DMSO (control) at room temperature for one hour. Each sample was subsequently proteolyzed with 2 μL 1:100 Pronase at room temperature for 32 min. To stop proteolysis, 3 μL cold 20 × Protease inhibitor was added to each sample, mixed, and placed on ice. The digested peptides were filtered through Vivacon 500 10 K spin column, precipitated using acetone, reduced with TCEP, alkylated with NEM, and digested with trypsin. Digests were desalted and used for LC–MS/MS data acquisition on an Orbitrap Lumos mass spectrometer (Thermo Fisher Scientific) coupled with an UltiMate 3000 RSLC-nano HPLC (Thermo Fisher Scientific) in data-dependent acquisition (DDA) mode.

### Xenograft and lung metastases mouse models

Mice experiments were approved by the National Cancer Institute (NCI) Animal Use and Care Committees. NOD-*Prkdc*^scid^*Il2rg*^*tm1Wjl*^ (NSG) mice (6–8 weeks old) or nude mice (6–8 weeks old) were obtained from NCI-Frederick animal facility and Jackson lab (Bar Harbor, ME, USA). C57BL/6 mice (4–5 weeks old) or BALB/c mice (4–5 weeks old) were purchased from Charles River Laboratories (Wilmington, MA, USA).

### Statistics

Data were presented as the mean and standard deviation (SD) or standard error of the mean (SEM), as indicated. Survival curves were generated using the Kaplan–Meier estimate and were compared using log-rank test. Other variables were analyzed using two-way ANOVA or unpaired Student’s t-test, as appropriate. Statistical analyses were performed using GraphPad Prism 6 (GraphPad Software, San Diego, CA). A *p* < 0.05 was considered as statistically significant.

## Results

### Carrimycin structure and synthesis

**Fig. 1:**
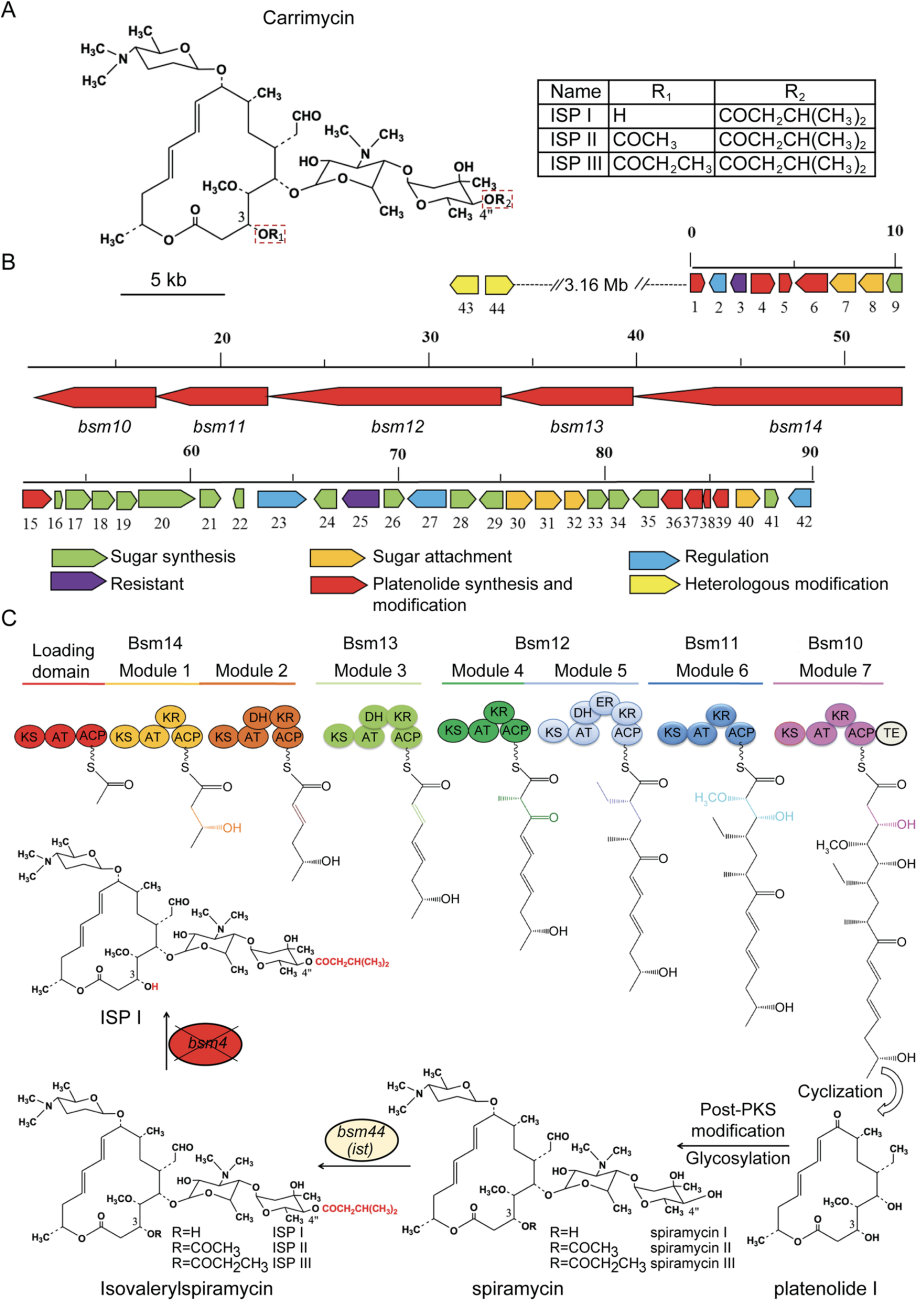
Genetic organization of the Carrimycin biosynthetic gene cluster and main steps of ISP I biosynthesis. **A** The chemical structure of Carrimycin and its main components: ISP I, II and III. **B** Genetic organization of the isovalerylspiramycin biosynthetic gene cluster. The proposed functions of the gene products in isovalerylspiramycin biosynthesis are indicated by various filling patterns. **C** Proposed pathway for ISP I biosynthesis. *bsm44* is 4″-*O*-isovaleryltransferase gene (*ist*) responsible for the formation of isovaleryl group at 4″ position. The *bsm4* is 3-*O*-acyltransferase gene involved in the acylation at 3″ position

Carrimycin, also known as bitespiramycin and shengjimycin, was approved recently by the China National Medical Products Administration. It is produced by recombinant *Streptomyces spiramyceticus*, which harbors a 4″-*O*-isovaleryltransferase gene (*ist*) from *Streptomyces thermotolerans* [2, 20]. Carrimycin’s composition is heterogenous and mostly consists of 4″-*O*-isovalerylspiramycins (ISP) I, II, III and trace amounts of 4″-*O*-acylspiramycin components (Fig. 1A).

Genomic sequencing of the Carrimycin-producing bacterial strain revealed a biosynthetic gene cluster of approximately 90 kb (Genbank accession number MH460451) (Fig. 1B). The gene cluster has a Spiramycin biosynthetic gene bundle as well as heterologous *ist* genes for 4″-*O*-isovalerylation of Spiramycin. Carrimycin’s structural backbone is a polyketide, putative platenolide I including ethylmalonyl-CoA, methylmalonyl-CoA and methoxymalonyl-CoA, which depends on polyketides synthase (PKS) and malonyl-CoA for its biochemical composition. After several post-PKS tailoring steps, including glycosylation, oxidation, acylation, and isovalerylation, a heterogenous mixture of ISP I, II and III results, differing only by acyl substitutions of the hydroxyl group on carbon 3 (Fig. 1C).

ISP I was isolated and purified from Carrimycin to a purity level of 98.47%, as detected by HPLC on ODS column (Fig. S1A). Its chemical structure was elucidated by spectra methods (Fig. S1B-H). The molecular formula C_48_H_82_N_2_O_15_ was established by HRMS spectrometry at *m/z* 927.5800 [M + H]^+^ (calculated 927.5788) (Fig. S1C). The NMR data were collected by Bruker Avance III. According to the ^1^H-NMR (Fig. S1D, Supplementary Table 1), ^13^C-NMR (Fig. S1E, Supplementary Table 1), ^1^H-^1^H cosy (Fig. S1F), and HSQC spectra (Fig. S1G), there is a sixteen-membered macrolide skeleton in ISP I along with an isovaleryl moiety and three deoxyhexoses forosamine, mycaminose and mycarose. The HMBC correlations indicated that the forosamine is connected to C-9, the mycaminose is connected with C-5, and the mycarose is connected to mycaminose with (1 → 4) glycosidic bond (Fig. S1H, Supplementary Table 1). The isovaleryl moiety is connected to C-4′′ of mycarose based on the correlation between 4.45 (m, 1H, H-4′′) and 172.09 (C-1′′′′) in HMBC spectrum. Therefore, the structure of ISP I was unambiguously assigned as 4′′-*O*-isovalerylspiramycin I (Fig. S1B).

### ISP I suppresses tumorigenesis and metastasis

**Fig. 2:**
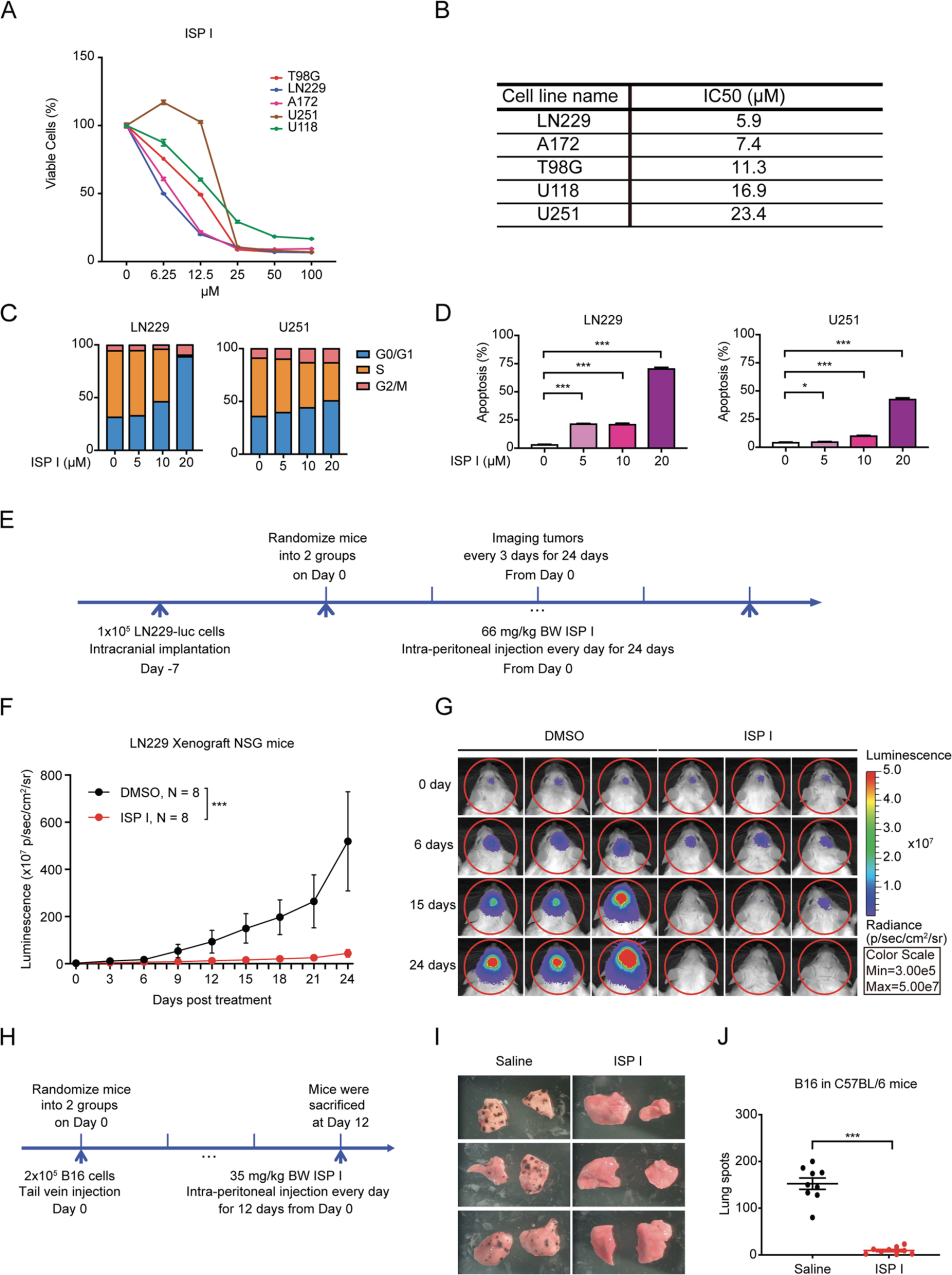
ISP I suppresses tumor growth and reduces lung metastases. **A** Dose–response curve of cell viability was measured by CCK-8 assay in five glioblastoma cell lines (T98G, U118, A172, LN229, and U251) treated with ISP I for 48 h. **B** IC50 values for the ISP I-treated glioblastoma cells. **C** and **D** Cell-cycle (**C**) and Annexin-V apoptosis (**D**) analysis in ISP I-treated LN229 cells and U251 cells. Cells were treated with ISP I for 6 h (**C**) and 48 h (**D**), separately. Summarized results from 4 independent wells are shown. **E** The schematic outline of the glioblastoma (LN229-luc) xenograft mouse model experiment. NSG mice were randomized into 2 treatment groups: DMSO (Control) (*N* = 8) and ISP I (*N* = 8). **F** Bioluminescence imaging was used to follow tumor progression. The luminescence signal demonstrated reduced tumor burden in the ISP I treatment arm compared to the DMSO treatment arm. ***p < 0.001 by two-way ANOVA. **G** Representative bioluminescence imaging of LN229-bearing mice. Three mice in the ISP I treatment arm demonstrated complete regression of tumor 24 days after the start of treatment. **H** The schematic outline of the melanoma (B16) lung metastasis mouse model experiment. C57BL/6 mice were randomized into 2 treatment groups: saline (Control) (*N* = 9) and ISP I (*N* = 9). **I** Representative lungs of mice corresponding to the ISP I and saline treatment arms. **J** Quantification of lung tumor nodules. All data are shown as mean ± SEM. *P* value: *p < 0.05; ***p < 0.001

To assess potential cytotoxicity, we treated glioblastoma cell lines with serial doses of Carrimycin main components, ISP I, II and III and assessed cell viability; the 50% inhibitory concentration (IC50) values showed that ISP I was most potent (Fig. 2A and B, and Fig. S2A to D). We focused our attention on ISP I.

Multiple tumor cell lines, including glioblastoma, renal cell carcinoma (RCC), and meningioma were sensitive to ISP I’s cytotoxic effect (Fig. 2A and B, and Figs. S4A and B, and S6A and B). Distribution across the cell cycle and apoptosis was assessed by flow cytometry, followed by EdU/DAPI or Annexin V/PI staining, separately. Flow analysis showed ISP I caused cell cycle arrest and induced dose-dependent apoptosis (Fig. 2C and D, and Figs. S3A and B, and S4C to F). Moreover, immunoblotting showed that cleaved PARP, a marker of apoptosis, increased in ISP I-treated LN229 cells and U251 cells (Fig. S3C). Likewise, ISP I increased cleaved caspase 3 and Bax but reduced Bcl-2 in a dose dependent manner (Fig.S3C). Furthermore, transcriptome profiling and Gene Set Enrichment Analysis (GSEA) of ISP I-treated LN229 cells both demonstrated ISP I was able to promote cell apoptosis (Fig. S3D and E).

We next investigated the tumor-suppressing effect of ISP I *in vivo*, using a human glioblastoma (LN229) xenograft intracranial mouse model (Fig. 2E). NSG mice were inoculated with luciferase-expressing cells in the right frontal cortex. After 7 days, intracranial tumor growth was confirmed with *in vivo* bioluminescence imaging; mice were randomized into ISP I or DMSO (control) groups. Mice treated with ISP I have significantly reduced tumor growth compared to controls (Fig. 2F and G). We confirmed these results in two additional xenograft models, using RCC (786-O) and meningioma (IOMM) cells (Fig. S5A to C and Fig. S6C to E). In all xenograft mouse models, ISP I treatment was well tolerated and animals maintained their bodyweight (Fig. S7A to C). Finally, we assessed ISP I’s anti-tumor effect in metastatic cancer. We examined two different, syngeneic murine lung metastases models: melanoma (B16) and mammary carcinoma (4T1). C57BL/6 mice were injected intravenously with B16 cells and randomized into ISP I-treated or saline-treated (control) groups (Fig. 2H). The lung metastasis mammary carcinoma model was established by injecting BALB/c mice with 4T1 cells into their second mammary fat pads. Seven days after tumor inoculation, BALB/c mice were randomized into ISP I-treated or saline-treated (control) groups (Fig. S8A). In both lung metastases models, ISP I significantly reduced lung tumor burden. After 12 days of ISP I treatment, B16-bearing mice had significantly reduced the number of lung metastases compared to saline-treated mice (Fig. 2I and J). Similarly, 4T1-bearing mice treated with ISP I had significantly fewer lung tumor nodules after 49 days of treatment compared to saline-treated mice (Fig. S8B and C). Collectively, these findings demonstrated that ISP I inhibited tumor growth *in vitro* and, more importantly, exerts potent and lasting antitumor effects in several different primary and metastatic tumors.

### ISP I targets to selenoprotein H

**Fig. 3:**
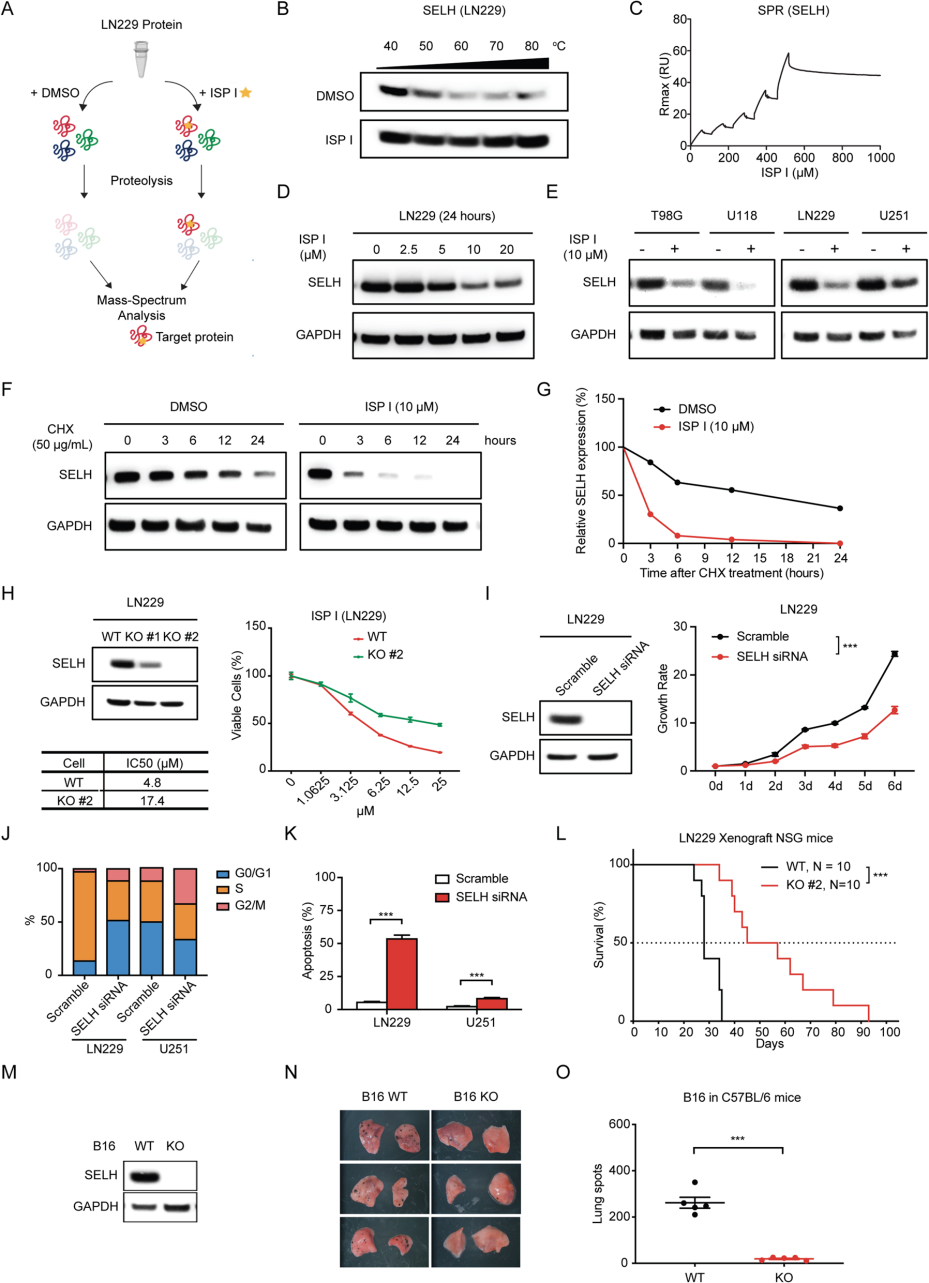
ISP I targets to SELH. **A** Scheme of a drug affinity responsive target stability (DARTS) assay. **B** The western blots corresponding to thermo-stability assay of ISP I binding to SELH extracted from LN229 cells. DMSO serves as a control. **C** Surface plasmon resonance (SPR) analysis of the interaction of ISP I and SELH synthesized in bacteria. **D** Western blots of SELH in LN229 cells treated with ISP I for 24 h. **E** Western blots of SELH in glioblastoma cell lines (T98G, U118, LN229, and U251) treated with ISP I (10 μM) for 24 h. **F** and **G** Cycloheximide (CHX) pulse chase assay shows SELH protein half-life in ISP I-treated LN229 cells by western blots. Quantification of the western blots was listed in G**. H** Western blots (left) show SELH expression in SELH knockout (KO) LN229 cells, KO #1 and KO #2. Dose–response curve of cell viability was measured by CCK-8 assay (right) in SELH-deficient LN229 cells (KO #2) or wild-type LN229 cells (WT). IC50 was calculated and listed. **I** Western blots show expression of SELH in the LN229 cells two days after SELH siRNA transfection. CCK-8 assay was performed to measure cell proliferation. LN229 cells transfected with scramble siRNA serve as a control. ***p < 0.001 by two-way ANOVA.** J** and **K** Cell-cycle (**J**) and Annexin-V apoptosis (**K**) analysis in SELH-deficient LN229 and U251 cells. Summarized results from 4 independent wells are shown. **L** Survival analysis of xenograft NSG mice intracranially inoculated with 3 × 10^5^ SELH-deficient LN229 cells (KO #2) or wild-type LN229 cells (WT). Median survival of KO #2 vs WT inoculated mice: 51 vs 28 days, ***p < 0.001 by log-rank test. *N* = 10 for per group. **M** Western blots show undetectable expression of SELH in B16 cells. **N** C57BL/6 mice received tail vein injection of 2 × 10^5^ wild-type or SELH-deficient B16 cells (*N* = 5 per group). Representative lungs of mice corresponding to wild-type or SELH-deficient B16 cells. **O** Quantification of lung tumor nodules. Expression of GAPDH serves as an internal control in (**D**, **E**, **F**, **H**, **I** and **M**). All data are shown as mean ± SEM. *P* value: ***p < 0.001

Given ISP I’s cytotoxicity across a variety of tumor cell lines, we sought to identify ISP I’s molecular target. We performed drug affinity responsive target stability (DARTS) assays in LN229 cells (Fig. 3A shows assay strategy). In DARTS, when ISP I bound a target protein, it created a stable conformational structure that inhibited proteases. Mass spectrum analysis showed that selenoprotein H (SELH), a thioredoxin-like protein with glutathione peroxidase activity [7, 21], was one of the most abundant primary protein presenting in ISP I-treated LN229 cells (Supplementary Table 2). Using a thermo-stability assay, we showed that ISP I protected SELH over a range of increasing temperatures (Fig. 3B and Fig. S9A), suggesting that ISP I targeted SELH.

Peroxidases are conserved enzymes catalyzing redox reactions that reduce hydroperoxides. They include several families with different active redox centers containing selenocysteine residues (e.g., selenoprotein), cysteine thiols (e.g., thioredoxin and thiol peroxidase), or heme cofactors (e.g., catalase) [22]. SELH has a conserved CXXU motif with redox function (cysteine separated by two other residues from selenocysteine) corresponding to the CXXC motif in thioredoxins [7]. To verify the specificity of ISP I’s targeting of SELH, we designed a surface plasmon resonance (SPR) assay to assess the interaction of ISP I with peroxidases synthesized in bacteria, including SELH, Thioredoxin (TrxA), Catalase (KatA), and Thiol peroxidase (TpX). ISP I tightly bound to SELH but not the other peroxidases (Fig. 3C and Fig. S10A to C). ISP I reduced SELH protein expression in a dose-dependent manner in multiple tumor cell lines (Fig. 3D and E, and Fig. S9B). Cycloheximide (CHX) chase assay confirmed that treatment with ISP I decreased SELH protein half-life, which suggested that ISP I promoted SELH protein degradation (Fig. 3F and G). Collectively, these results showed that ISP I targeted SELH and promoted SELH protein degradation.

To confirm that ISP I inhibits cell growth via a SELH-dependent mechanism, we generated SELH-deficient LN229 and RCC cell lines (786-O and RCC4, respectively) with CRISPR-Cas9. SELH-deficient cells resist ISP I treatment when compared to wild-type cells (Fig. 3H and Fig. S9C). Using selective siRNA knock-down, SELH inhibition significantly decreased cell growth rate, retards cell proliferation, and increased apoptosis (Fig. 3I to K, and Figs. S9D to G, and S11A and B). To evaluate whether SELH regulates tumor growth *in vivo*, NSG mice were orthotopically injected with either wild-type or SELH-deficient LN229 cells. Mice implanted with SELH-deficient LN229 cells have an almost twofold increase in median overall survival compared to mice with wild-type LN229 cells (control) (SELH-deficient LN229 group vs wild-type LN229 group: 51 vs. 28 days, Fig. 3L). SELH’s regulatory tumor growth effect was validated in the melanoma lung metastasis model. C57BL/6 mice were injected with either wild-type B16 cells or SELH-deficient B16 cells (Fig. 3, M to O). Twelve days after tail-vein inoculation, mice with SELH-deficient B16 cells have significantly fewer lung tumors when compared to mice injected with wild-type B16 cells (Fig. 3N and O). Thus, ISP I inhibited growth of primary and metastatic tumors through inhibition of SELH.

### ISP I disrupts intracellular redox homeostasis

**Fig. 4:**
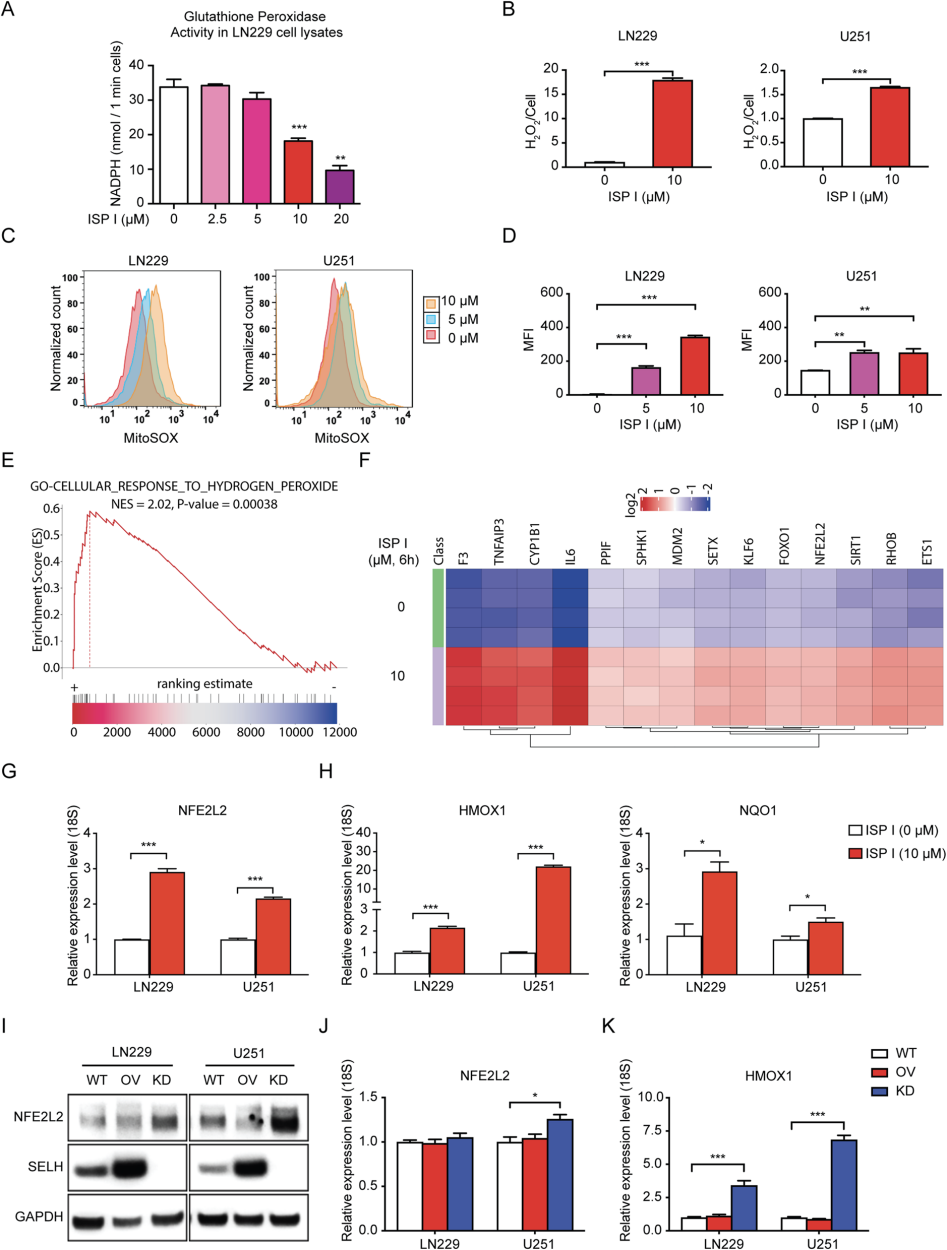
ISP I triggers ROS accumulation and oxidative stress response pathways. **A** Glutathione peroxidase activity assay shows a dose-dependent decrease of anti-oxidative enzyme activity in LN229 cells treated with ISP I. **B** Quantification of intracellular reactive oxygen species (ROS) levels by ROS-Glo H_2_O_2_ assay. LN229 and U251 cells were treated with ISP I at 10 μM for 24 h. All groups were normalized to saline-treated groups. **C** Flow cytometry analysis of ROS level using MitoSOX staining in ISP I-treated LN229 and U251 cells. Cells were pre-treated with 5 μM or 10 μM ISP I for 24 h. **D** Summarized ROS levels by mean fluorescence intensity (MFI) of MitoSOX positive cells from 3 independent wells are shown. **E** and **F** Transcriptomic profiling reveals that ISP I provokes an oxidative stress response pathway. **E** Gene Set Enrichment Analysis (GSEA) showing that ISP I-treated cells are highly enriched in genes associated with oxidative stress response. NES = 2.02. *P* < 0.05. **F** Heatmap of statistically significant differential gene expression as determined by RNA-seq between saline-treated and ISP I-treated cells. LN229 Cells were treated with ISP I at 10 μM or saline for 6 h. *N* = 4. *P* < 0.05. **G** Real-time RT-PCR analysis shows *NFE2L2* mRNA expression in glioblastoma cell lines (LN229 and U251) treated with 10 μM ISP I for 24 h. 18S expression serves as an internal control**. H** Real-time RT-PCR results show the mRNA expression of NFE2L2 downstream gene-*HMOX1* and *NQO1* with 18S as an internal control. **I** Western bots show NFE2L2 and SELH protein expression in LN229 and U251 cells with SELH overexpression or with knocking down of SELH. GAPDH expression was used as an internal control. **J** and **K** Real-time RT-PCR results show *NFE2L2* mRNA (**J**) and its downstream gene *HMOX1* mRNA (**K**) in SELH knockdown LN229 and U251 cells. All data are shown as mean ± SEM. *P* value: *p < 0.05; **p < 0.01; ***p < 0.001

Because selenoproteins can protect against reactive oxygen species [5, 6], we investigated whether ISP I altered redox homeostasis *in vitro*. First, we assessed ISP I’s effect on glutathione peroxidase activity. ISP I-treated LN229 cells exhibit significantly reduced glutathione peroxidase activity (Fig. 4A). ROS-Glo H_2_O_2_ assay confirms that ISP I-treated tumor cells have increased intracellular ROS levels (Fig. 4B and Fig. S12A). MitoSOX staining, followed by flow cytometry, shows that ISP I-treated cells generate higher intracellular ROS, in a dose-dependent manner (Fig. 4C and D, and Fig. S12B and C).

We assessed whether ISP I alters antioxidant downstream signaling pathways. Transcriptome profiling and GSEA of ISP I-treated LN229 cells both demonstrated widespread changes in oxidative pathways as well as specific upregulation of the nuclear factor erythroid 2-related factor 2 (NFE2L2/NRF2) (Fig. 4E and F), a transcription factor that regulates redox homeostasis by binding to the regulatory regions of antioxidant response elements (ARE) [23]. *NFE2L2* expression was significantly upregulated in ISP I-treated cells; ISP I induced an increase in the downstream targets of NFE2L2 signaling, *HMOX1* and *NQO1* (Fig. 4G and H, and Fig. S12D and E). We measured NFE2L2 expression in glioblastoma cells that either overexpressed SELH or had SELH expression silenced. Overexpression of SELH decreased NFE2L2 protein levels, while NFE2L2 proteins were increased in SELH-silenced cells (Fig. 4I). *NFE2L2* mRNA was slightly increased in SELH-silenced U251 cells (Fig. 4J). Of note, SELH-silenced cells had a significant increase in *HMOX1* mRNA, consistent with an increase in NFE2L2 protein (Fig. 4K). By suppressing SELH, ISP I induced ROS accumulation and activated antioxidative signaling, including the NFE2L2 pathway.

### ISP I triggers DNA damage and R-loop formation

**Fig. 5:**
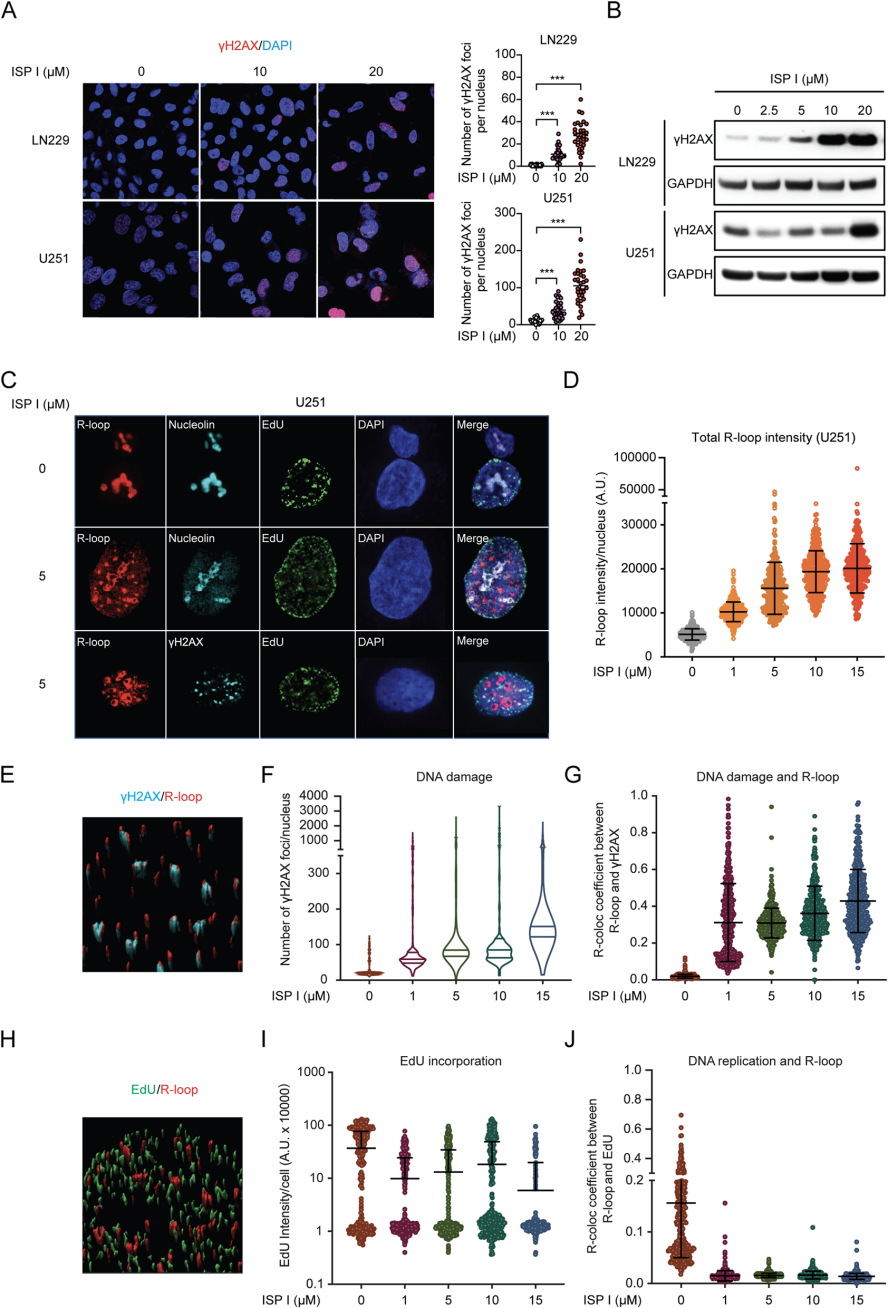
ISP I triggers DNA damage, R-loop formation, and alters the localization of R-loops. **A** and **B** Immunofluorescence staining assay (**A**) and western blots (**B**) show γH2AX expression in LN229 and U251 cells treated with ISP I at indicated concentrations for 6 h. The number of γH2AX foci in cell nucleus was identified and quantified. Expression of GAPDH serves as an internal control. **C** and **D** Immunofluorescence staining assay shows R-loop formation in U251 cells. U251 cells were transfected with V5-tagged catalytically dead RNaseH1 for recognition of R-loops, and then treated with ISP I at indicated concentrations for 30 min. U251 cells were co-stained with antibodies recognizing R-loop (V5, red), DNA replication (EdU, green), nucleolus (nucleolin, blue) or DNA damage (γH2AX, blue). Cell nuclei were counterstained with DAPI (purple). Representative immunofluorescence staining images were shown in (**C**). Quantitative comparations of ISP I’s effect on R-loop formation are shown in (**D**). **E** Representative images show colocalization of R-loops (red) with γH2AX (blue). **F** Quantitative comparations of ISP I’s effect on DNA damage show an increase in γH2AX foci formation in U251 cells. **G** The number of R-loops on DNA damage sites were identified and counted by the colocalization of R-loops with γH2AX. **H** Representative image shows no colocalization of R-loops (red) with EdU (green). **I** Quantitative comparisons of ISP I’s effect on EdU incorporation show a reduction in EdU incorporation in U251 cells. **J** The number of R-loops on DNA replication sites were identified and quantified by the colocalization of R-loops with EdU. All data are shown as mean ± SEM. *P* value: *p < 0.05; **p < 0.01; ***p < 0.001

Since increased intracellular levels of ROS cause oxidative DNA damage, which induces genomic instability and inhibits cell cycle progression [24], we assessed whether treatment with ISP I augmented DNA damage in cancer cells. Immunofluorescence staining and western blot analyses revealed that ISP I-treated cells showed higher quantities of γH2AX, a sensitive marker of DNA damage (Fig. 5A and B, and Fig. S12F and G). To further understand the role of ISP I-induced ROS in DNA damage, we performed Immunofluorescence staining and western blot analyses for γH2AX in ISP I-treated LN229 cells and U251 cells with the presence of N-acetylcysteine (NAC), a ROS scavenger. Both analyses showed that supplementing NAC (10 mM) rescued increased γH2AX to the basic level of that in the cells without ISP I treatment (Fig. S13A to C), suggesting that ISP I-induced DNA damage was due to elevated ROS generation.

To assess ISP I’s effect within the nucleus, we evaluated R-loop formation in cancer cells. R-loops are DNA-RNA hybrid structures composed of a displaced single-stranded DNA hybridized with the nascent RNA transcript [25]. R-loops are strongly induced by ROS within the nucleus and can trigger DNA damage, genome instability, and cell cycle arrest [26]. We quantified R-loop formation using a human bone osteosarcoma cell line (U2OS) or a glioblastoma cell line (U251) overexpressing the V5-tagged catalytically-inert RNase H (R-loop marker), a dynamic strategy for R-loop profiling [27]. ISP I-treated cells demonstrated increased R-loop accumulation in a dose-dependent fashion (Fig. 5C and D, and Fig. S14A and B). To assess DNA replication, we stained tumor cells with 5-ethynyl-2′-deoxyuridine (EdU). ISP I reduced EdU incorporation into DNA in a dose-dependent manner (Fig. 5C and I, and Fig. S14A and C). Co-immunofluorescence staining with antibodies specific for nucleolin (a marker of nucleolus), γH2AX and EdU revealed that treatment with ISP I not only consistently increased DNA damage and reduced DNA replication but also altered R-loop localization (Fig. 5C, E to J). R-loop foci concentrated in nucleoplasm instead of the nucleolus in ISP I-treated U251 cells (Fig. 5C). R-loop foci specifically localized at DNA damage sites, not DNA replication sites (Fig. 5G and J). Furthermore, we saw the delocalization of nucleolin in ISP I-treated U251 cells, evidence of nucleolar stress (Fig. 5C). Collectively, ISP I significantly augmented intracellular levels of ROS, which decreased DNA replication and led to DNA damage and R-loop formation.

### ISP I impairs nucleolar rRNA transcription via JNK2/TIF-IA pathway

**Fig. 6:**
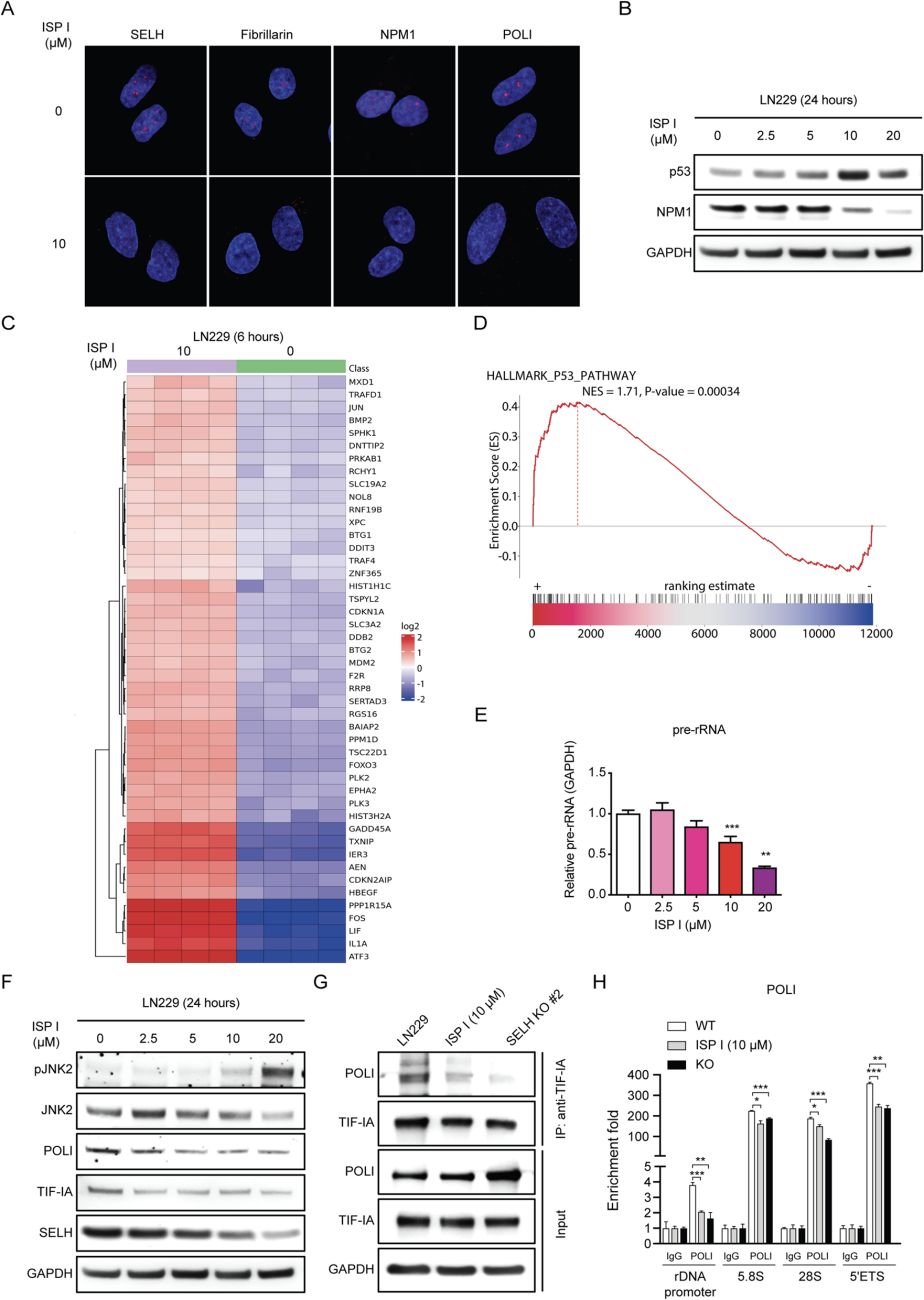
ISP I induces a nucleolar stress response. **A** Immunofluorescence staining assay shows a loss of SELH and nucleolar labelling of Fibrillarin, NPM1 and POLI, in ISP I-treated cells. LN229 cells were treated with ISP I at 10 μM for 8 h. **B** Western blots show NPM1 and p53 expression in LN229 cells treated with ISP I for 24 h. GAPDH expression was used as an internal control. **C** and **D** Transcriptomic profiling reveals that ISP I activates p53 pathway**. C** Heatmap of statistically significant differential gene expression as determined by RNA-seq between saline-treated and ISP I-treated cells. LN229 cells were treated with ISP I at 10 μM or saline for 6 h. *N* = 4. *P* < 0.05. **D** GSEA demonstrates that ISP I-treated cells are highly enriched in genes associated with p53 activation. NES = 1.71. *P* < 0.001. **E** Real-time RT-PCR analysis shows pre-rRNA transcription in LN229 cells treated with ISP I for 8 h. GAPDH expression was used as an internal control. **F** Western blots show phosphorylated JNK2, POLI, and TIF-IA expression in LN229 cells treated with ISP I for 24 h. GAPDH expression was used as an internal control. **G** Immunoprecipitation by anti-TIF-IA antibody showing POLI interacts with TIF-IA in LN229 cells. The POLI and TIF-IA interaction is interrupted in ISP I-treated (10 μM, 24 h) and SELH-deficient LN229 cells. **H** Quantitative CHIP evaluations showing that ISP I treatment (10 μM, 24 h) or deficiency of SELH reduces the occupancy of POLI on the promoter and the coding region (5’ETS, 5.8S and 28S) of rDNA. All data are shown as mean ± SEM. *P* value: *p < 0.05; **p < 0.01; ***p < 0.001

We investigated ISP I’s ability to induce the nucleolar stress response. LN229 cells were immunostained with primary antibodies against nucleolar proteins: Fibrillarin, Nucleophosmin (NPM1), and RNA Polymerase I (POLI). Immunofluorescence imaging revealed increased nucleolar protein dispersion in ISP I-treated LN229 cells and a pronounced decrease in SELH expression within the nucleolus (Fig. 6A). NPM1 is a critical nucleolar protein involved in several key regulatory pathways, including mRNA transport, ribosomal biogenesis, chromatin remodeling, apoptosis, and genome instability [10, 28]. NPM1 protein level fell and p53 (an apoptotic marker) increased in ISP I-treated LN229 cells in a dose-dependent manner (Fig. 6B). GSEA profiling confirmed the induction of the p53 pathway (Fig. 6C and D).

To assess ribosomal biogenesis alterations and probe potential links with nucleolar dysfunction, we analyzed pre-rRNA transcription. ISP I reduced pre-rRNA (POLI transcript) in a dose-dependent manner (Fig. 6E). The quantities of mature 18S RNA (rRNA cleavage) and pre-GAPDH mRNA (POL II transcript) were not altered (Fig. S15A); conversely, pre-tRNA 13 (POL III transcript) and some mature tRNAs (tRNA) were increased (Fig. S15A and B). Stress-dependent inhibition of POLI transcription is mediated through inactivation of TIF-IA, a POLI-specific co-activator, via specific phosphorylation of TIF-IA at a single threonine residue (Thr 200) by JNK2 [11]. Phosphorylation at Thr 200 impairs the interaction of TIF-IA with POLI thereby abrogating initiation complex formation and rRNA synthesis [11]. JNK2 belongs to the family of stress-activated protein kinases that play a crucial role in the cellular response to oxidative stress [29]. ISP I increased JNK2 phosphorylation in a dose-dependent manner, indicating elevated cellular ROS-induced JNK2 activation (Fig. 6F). Although ISP I-treatment led to a slightly decreased trend in POLI and TIF-IA expression, co-immunoprecipitation assays showed that the physiological interaction between TIF-IA and POLI was significantly disrupted in ISP I-treated or SELH-deficient LN229 cells (Fig. 6G), suggesting that activated JNK2 mainly impaired TIF-IA and POLI interaction in SELH-deficient cells. To confirm reduced POLI initiation complex formation and rRNA transcription in SELH-deficient cells, chromatin immunoprecipitation (CHIP) assay was performed in ISP I-treated or SELH-deficient LN229 cells. CHIP assay showed reduced POLI recruitment to the promoter and the coding regions of rDNA (5.8S, 28S and 5’ETS) in ISP I-treated or SELH-deficient LN229 cells (Fig. 6H). This suggested that ISP I induced suppression of SELH impaired POLI-rDNA interaction by activating the ROS/JNK2/TIF-IA pathway. These findings demonstrated that ISP I disrupted ribosomal biogenesis by suppression of POLI transcription but does not alter the mature RNA machinery.

## Discussion

Macrolide antibiotics are traditionally used as broad-spectrum antibacterial reagents against Gram-positive bacteria. Many of them, such as rapamycin [30], clarithromycin [31], and epothilone[32], have been reported to have anti-cancer potency and proposed as anti-cancer drugs for various tumors.

Carrimycin, a newly-developed macrolide derivative synthesized with recombinant DNA technology, has demonstrated cytotoxicity in various cancer cells. Carrimycin may exert its function through PI3K/AKT/mTOR pathway and MAPK pathway in oral squamous cell carcinoma cells, while suppressing tumor growth of hepatocellular carcinoma by inhibition of VEGF and PD-L1 protein expression [3, 4]. However, the direct target and molecular mechanism of Carrimycin in inhibiting tumor progression was unclear. Using DARTS and mass spectrum analyses, we identified that the active component of Carrimycin-ISP I bound to and promoted SELH protein degradation, which may play an important and novel role in cancer treatment.

Cycloheximide (CHX) chase assay showed that ISP I decreased SELH protein half-life, which suggested that ISP I promoted SELH protein degradation (Fig. 3F and G). SELH is a selenoprotein, which can be degraded through ubiquitin-activated proteasomal pathways or the lysosome-mediated pathway [33]. Previous studies reported that directly appending a hydrophobic tag to the surface of a protein would mimic the partially denatured state of the protein, thus inducing its proteasomal degradation [34]. ISP I, a hydrophobic macrolide, may directly interact with SELH to induce degradation in the manner of hydrophobic tagging. In addition, Cullin-RING-E3 ubiquitin ligase 2 (CRL2) targets the N-terminal of selenoproteins for ubiquitin-proteasomal degradation [35]. ISP I may crosslink SELH and CRL2 to induce ubiquitylation and degradation of SELH. We believe the binding structure of ISP I and SELH either changed the protein folding or modulated ubiquitin ligase substrate selectivity, leading to SELH degradation. The detailed mechanism awaits further investigation.

Intriguingly, deficiency of SELH in zebrafish disrupts redox homeostasis and induces ROS generation [36]. While normal cells balance redox homeostasis carefully, transformed cells have typically developed additional, adaptive responses to protect themselves from the hazardous effects of oxidative stress. However, they remain susceptible to exogenous ROS, which could be exploited for cancer therapy [37, 38]. To this end, ISP I inhibits SELH and enhances intracellular ROS generation and promotes oxidative response signaling.

Another common feature of malignant cells is the elevation of ribosomal RNA (rRNA) levels, with concomitant nucleolar hypertrophy [12]. Agents targeting the nucleolus to inhibit rRNA synthesis will arrest cancer cell proliferation [39]. For example, inhibition of the rate-limiting enzyme for *de novo* guanine nucleotide biosynthesis (IMP dehydrogenase-2) suppresses glioblastoma [40]. CX-5461, which restrains RNA POLI and suppresses tumorigenesis by hampering rRNA synthesis [41]. BMH-21, which degrades RNA POLI, induces cell death [37]. ISP I, however, modulates POLI transcriptional function by inducing nucleolar endogenous oxidative stress, to which cancer cells are susceptible. Additionally, ISP I induces R-loop formation outside the nucleolus at sites of DNA damage; impairs rRNA transcription in a dose-dependent fashion, so that NPM1 protein level falls and p53 rises; and substantially enhances ROS activity through the JNK2/TIF-IA pathway, all of which induce nucleolar dysfunction and enhance sensitivity to ROS in tumor cells.

Finally, ISP I inhibits both local growth and distant spread in several different primary and metastatic tumors. Elevated levels of ROS suppress tumor angiogenesis and metastasis by destroying cancer cells, whereas lower, non-lethal concentrations assist cancer metastasis [38]. Recent evidence indicates that nucleoli play key roles not only in tumorigenesis but also in metastasis [39]. Metarrestin, which disrupts nucleolar structure and restricts POLI transcription, suppresses metastasis in mouse models of human cancer [42]. Overexpression of ribosomal protein RPL15 promotes breast cancer metastasis [39]. These reports buttress the idea that the nucleolus plays a crucial role in tumorigenesis and metastasis [36].

## Conclusions

In conclusion, a novel, recombinant macrolide antibiotic exerts a potent anti-cancer effect through inhibition, in the nucleolus, of a specific protein, SELH. This creates vulnerability in cancer cells by disrupting RNA polymerase I and inducing subtle but catastrophic DNA damage, as well as by causing cell cycle arrest and tumor cell apoptosis. ISP I, which has minimal deleterious effects on normal tissues in mice, targets SELH specifically. By exploiting SELH inhibition, which links derangements in the ROS homeostasis with ribosomal synthesis dysfunction, ISP I reveals a unique vulnerability in neoplastic cells. Thus, the nucleolus and SELH are promising targets for cancer therapy.

## Supplementary Information

**Additional file 1.**

## Data Availability

The datasets used and/or analyzed during the current study are available from the corresponding author upon reasonable request.

## Abbreviations

ISP I: Isovalerylspiramycin I
SELH: Selenoprotein H
DARTS: Drug affinity responsive target stability
ROS: Reactive oxygen species
POLI: RNA polymerase I
JNK2: C-Jun N-terminal kinase
TIF-IA: Transcription initiation factor IA
rRNA: Ribosomal RNA
IC50: 50% Inhibitory concentration
RCC: Renal cell carcinoma
SPR: Surface plasmon resonance
TrxA: Thioredoxin
KatA: Catalase
TpX: Thiol peroxidase
CHX: Cycloheximide
GSEA: Gene Set Enrichment Analysis
NFE2L2/NRF2: Nuclear factor erythroid 2-related factor 2
ARE: Antioxidant response elements
EdU: 5-Ethynyl-2′-deoxyuridine
NPM1: Nucleophosmin
NAC: N-acetylcysteine
CRL2: Cullin-RING-E3 ubiquitin ligase 2
