# Supplementary material for: Targeting selenoprotein H in the nucleolus suppresses tumors and metastases by Isovalerylspiramycin I
Source: J Exp Clin Cancer Res. 2022 Apr 6;41:126. doi: 10.1186/s13046-022-02350-0 (PMC8985259; doi:10.1186/s13046-022-02350-0)
Supplement: Supplementary file 1 — Additional file 1. [file 13046_2022_2350_MOESM1_ESM.pdf]

Targeting selenoprotein H in the nucleolus suppresses tumors and metastases by Isovalerylspiramycin I

Supplementary Methods

Methods

Cell culture and reagents

LN229-luc cells were generated by stable transfection of luciferase-containing lentivirus (EF1a-ffLuc2-eGFP) into LN229 cells and stable monoclonal cells were selected with puromycin (Sigma).

U251 cells or U2OS cells were transfected with plasmid containing RNaseH1 construct mutated at D210N (Addgene #111904) and WKKD (Addgene #111905). Stable monoclonal cells were selected with hygromycin.

SELH-knockout cell lines were generated using the CRISPR-Cas9 technique as previously described [1]. The gDNA for targeting human SELH in LN229 cells was designed as follows: Oligo 1, 5'- GCCTTACGCTTCCTCCCGCG -3'; Oligo 2, 5'-CTCGGCTACGGCGACCACCG -3', The gDNA for targeting mouse SELH in B16 cells was designed as follows: Oligo 1, 5'- GTAAGGCGGGGGCCGCGCCTA -3'; Oligo 2, 5'- GCGCCTTACGCTTTCTTCCGT -3', and subcloned into Cas9 carrying vector (pX330). The two resultant plasmids and puromycin expressing vector (pPGK-puro) were co-transfected at a ratio of 1:1:1 into LN229 or B16 cells using lipofectamine 2000 (Invitrogen). 24 hours later, the transfected cells were subjected to puromycin (Sigma) at 1 μg/mL for 7 days. One week after selection, the surviving clones were expanded and total protein for standard western blot analysis was extracted.

4T1 cells were cultured in RPMI-1640 medium (Gibco), supplemented with 10% fetal bovine serum (FBS; Gibco) and 1% penicillin and streptomycin (Gibco). All other cells were cultured in Dulbecco's Modified Eagle Medium (DMEM; Gibco).

Real-time RT-PCR

Total RNA was extracted using PureLink RNA Mini Kit (ThermoFisher Scientific, Waltham, MA, USA) according to the manufacturer's protocol. cDNA was synthesized with iScript cDNA Synthesis Kit (Bio-Rad, Hercules, CA, USA). For real-time PCR analysis, SsoAdvanced Universal SYBR Green Supermix kit (Bio-Rad, Hercules, CA, USA) was used according to the manufacturer's instruction. Reactions were performed using the pre-set program of a Bio-Rad CFX Real-time system (BioRad, Hercules, CA, USA) in a 384-well plate format. All primers used are shown in Supplementary Table 3.

RNA-seq

LN229 cells were treated with DMSO or ISP I at 10 μM for 6 hours, and RNA was extracted using the RNeasy plus micro kit (Qiagen) according to the manufacturer's instructions. Quality control was performed by Bioanalyzer (Agilent), and RNA samples with an RNA integrity number >9 were subsequently used. mRNA sequencing libraries were prepared using the SMARTer Ultra Low Input RNA kit v3 (Clontech) and Nextera XT DNA library preparation kit (Illumina). Paired-end sequence reads of 126 bp were generated by a HiSeq2500 sequencer (Illumina). The raw RNA-seq FASTQ reads were aligned to the human genome (hg19). Gene features were subsequently summarized using subread algorithm and differentially-expressed genes were derived using limma-voom method with quantile normalization. The gene- and sample-specific normalization factors were then used to correct counts. Gene Ontology and GSEA of biological processes were determined by GAGE (Gene Ontology Consortium) [2].

Immunoblotting

Cells were lysed by RIPA buffer from ThermoFisher Scientific and the concentration was measured using DC Protein Assay (Bio-Rad, Hercules, CA, USA). Equal amount of protein of each sample were loaded for gel electrophoresis and transferred to nitrocellulose membrane, blocked, and incubated overnight at 4 °C with primary antibodies. Membranes were then washed with TBST (3 times, 10 min each). Exposure, imaging, and data analysis were done using a KwikQuant Imager system (Kindle Biosciences, USA). The following antibodies were used: anti-phospho-histone H2A.X (Ser139) (Cell Signaling, #9718), anti-NFE2L2 (Cell Signaling, #12721), anti-SELH (Abcam, ab151023), anti-p53 (Millipore sigma, MABE327), anti-NPM1 (Cell Signaling, #3542), anti-phospho-JNK (Thr183/Tyr185) (Cell Signaling, #4668), anti-JNK2 (Cell Signaling, #9258), anti-POLI (Cell Signaling, #24799), anti-TIF-IA

(Abcam, ab112052), anti-cleaved PARP (Cell Signaling, #9541), anti-cleaved caspase 3 (Cell Signaling, #9661), anti-Bax (B&D, #666467), anti-Bcl-2 (B&D, #516517GR), anti-GAPDH (Millipore, MAB374).

Immunohistochemistry

For immunofluorescence assay, cells were seeded on a 35 mm confocal dish with glass bottom and treated with ISP I. After 24 hours, cells were fixed with 4% paraformaldehyde, blocked with 4% goat serum (Abcam), and labeled with primary antibodies overnight at 4 °C. Cells were then incubated with secondary antibodies conjugated with goat anti-rabbit Alexa Fluor-594 (ThermoFisher Scientific, A11037), followed by staining with DAPI for nuclei. Images were obtained using Zeiss LSM 780 microscopy (Carl Zeiss, Oberkochen, Germany). The following primary antibodies were used: anti-phospho-histone H2A.X (Ser139) (Cell Signaling, #9718), anti-SELH (Abcam, ab151023), anti-Fibrillarin (Cell Signaling #2639), anti-NPM1 (Cell Signaling, #3542), anti-POLI (Cell Signaling, #24799). The number of γH2AX foci in nucleus was counted using Image J software.

EdU and R-loop staining for U2OS cells

U251 cells or U2OS cells containing RNaseH1 construct mutated at D210N were incubated with EdU for 20min and washed with a brief PBS wash. These cells were then pre-extracted in PBS-T buffer (0.2% Triton X-100 in 1× PBS, phenylmethylsulphonyl fluoride [PMSF], protease inhibitor cocktail [Sigma, P8340] and phosphatase inhibitor cocktail [Roche, P4906845001]) for 3 min on ice and washed briefly with PBS. Pre-extracted cells were fixed using 2.0% paraformaldehyde (PFA) solution in PBS. EdU staining was performed as manufacturer's instructions. Primary antibody staining was performed as follows: anti-V5-tag (Santacruz, sc-271926, 1:250 dilution), anti-Nucleolin (Cell Signaling, 14574), anti-phospho-histone H2A.X (Ser139) (Cell Signaling, #9718). Secondary antibody staining was performed as follows: Alexa 568 conjugated anti-mouse IgG (1:500, Thermo Fisher Scientific), Alexa 647 conjugated anti-rabbit IgG (1:500, Thermo Fisher Scientific). A Zeiss LSM710 confocal microscope was used for imaging. For R-loop super-resolution microscopy, immunofluorescence slides were imaged with a VisiTech (Sunderland, UK) VT-iSIM super-resolution microscope, using a ×100 NA 1.45 Nikon PSF-optimized objective. Fluorophores were excited using the appropriate lasers, Diode 405 nm, Diode 488 nm, OPSL 561 nm, or

Diode 642 nm, and super-resolution emission was collected with a 16-bit CMOS camera (Hamamatsu, Japan). Z-sections were obtained at 100 nm intervals and deconvolved with the VisiTech proprietary software (Microvolution). Images were scored in IMARIS software by counting non-nucleolar R-loops foci where nucleoli were masked based on intensity and size of R-loop staining. Number of foci was counted in the rest of the nucleus.

Cell viability assay

Cell viability was measured by Cell Counting Kit-8 (CCK-8, Dojindo Molecular Technologies, Tokyo, Japan). Cells were seeded in 96-well plates at a density of 3×10^3 cells/well and cultured for 24 hours before treated with ISP I, ISP II or ISP III at different concentration. After treatment with DMSO/saline solutions (control) or with a gradient of ISP I concentrations ranging from 0 μM to 100 μM for 48 hours, cells in each well were incubated with 10 μL of CCK-8 solution for an additional 2 hours. The absorbance in each well at a wavelength of OD450 was detected by the Synergy H1 microplate reader (BioTek, Winooski, VT, USA).

Cell apoptosis and cell cycle

2×10^5 Cells were plated in 6-well plates and treated with different concentrations of ISP I. Cells were subsequently harvested and washed three times with PBS. Cells were resuspended in 100 μL binding buffer and incubated with 5 μL APC-conjugated Annexin V working solution (BD bioscience, Franklin Lakes, NJ, USA) and 1 μL propidium iodide (PI, Invitrogen, Waltham, MA, USA) for 15 min at room temperature and protected from light. Data acquisition and quantification were processed with BD LSRFortessa flow cytometer using FlowJo software (Ashland, OR, USA).

For monitoring cell cycle arrest of ISP I treated cells, Click-iT EdU Flow Cytometry Assay Kits (ThermoFisher Scientific, Waltham, MA, USA) were used. Cells were cocultured with EdU at a concentration of 10 μM for 1 hour. After fixation and permeabilization, EdU positive cells were labeled with Alexa Fluor 647 fluorescein. DAPI was used for measuring total DNA content for identifying different cell cycle phases. Data were collected using BD LSRFortessa flow cytometer using FlowJo software (Ashland, OR, USA). Cells that were positive with EdU and DAPI were categorized as being in the S-Phase of the cell cycle.

Analysis of mitochondria superoxide

To detect the ISP I-mediated mitochondrial changes in superoxide, we used MitoSOX Red mitochondrial superoxide indicator kit (ThermoFisher Scientific, Waltham, MA, USA). Cells were incubated with 5 μM of MitoSOX Red for 15 min at room temperature followed by flow cytometry analysis. The excitation of 561 nm laser and 610/20 discriminating filter were applied by BD LSRFortessa flow cytometer.

Cell ROS and glutathione-dependent peroxidases levels measurement

The ISP I-mediated changes in intracellular ROS level were measured by ROS-Glo H_2O_2 assay kit (Promega, Madison, WI, USA). After treatment with ISP I for 24 hours, H_2O_2 Substrate were added and incubated for 6 hours. The supernatant was collected and incubated with ROS-GloTM Detection Solution for 20 min at room temperature and protected from light. Luciferase data were obtained with the Synergy H1 microplate reader (BioTek, Winooski, VT, USA).

Glutathione peroxidase kit (Abcam, Cambridge, MA, USA) was used to quantify the activity of the glutathione-dependent peroxidases in live cells. 2×10^6 cells were harvested and homogenized with assay buffer provided in the kit. The supernatant was then collected and incubated per manufacturer's instructions. Absorbance at 340 nm was measured using the microplate reader mentioned previously.

Drug affinity responsive target stability (DARTS) assay

The database search and label-free quantitation were performed with Proteome Discoverer 2.4. The false discovery rate for peptide-spectrum matches (PSMs) was set to 0.01 using Percolator. Protein abundance used for ratio calculation was the sum of unique peptides matched to the protein. Total peptide amount was used for normalization. Background based t-test was used for hypothesis test.

Cellular thermal shift assay (CETSA)

CETSA experiments were performed to determine whether ISP I-related ligand induced thermal shifts. Intact and live cells in 10 cm dish were harvested, washed, and re-suspended in PBS with protease inhibitor cocktail. Cell proteins were extracted by subjecting cells to 5 freeze-thaw cycles in liquid nitrogen. The supernatant was centrifuged at 20, 000g for 20 min at 4 °C and samples were then aliquoted and incubated with different concentrations of ISP I for 1 hour. Samples were subsequently

aliquoted into PCR tubes and thereafter heated at gradient temperatures ranging from 40 °C to 80 °C for 3 min. Lastly, samples were centrifuged and resolved using 4-12% SDS-PAGE for western blot experiments.

Surface plasmon resonance (SPR) assay

The affinities constant (KD) and kinetics (ka and kd) of ISP I binding to SelH (Sec44→Cys44) were assessed using Biacore 8K (GE Healthcare, Sweden) at 25 ℃. Stock solution 10 x PBS-P+ (with 0.5% P20) provided by GE was used to prepare running buffers, 4-point solvent correction and samples for binding in 5% DMSO. The purified active SelH (Sec44→Cys44) was diluted by 10 mM sodium acetate solution at pH 5.5, resulting in a protein concentration of 50 μg/mL. Coupling conditions were determined by protein isoelectric points and previous test. The diluted protein was immobilized on the surface of a CM5 sensor chip via the primary amine group, employing a standard Amine Coupling Kit, and the target immobilization level was 7000 response units (RUs).

To determine the binding affinity between ISP I and SelH (Sec44→Cys44), a series of ISP I dilutions were analyzed by single-cycle kinetics. A concentration gradient of ISP I, as the analyte, was freshly prepared in PBS-P+ running buffer (with 5% DMSO) with at least five concentrations (31.25, 62.5, 125, 250, 500μM). Various gradient concentrations of ISP I, including a zero concentration (running buffer), were flowed over immobilized SelH (Sec44→Cys44), with 120 s for binding, followed by disassociation for 120 s, and the obtained response units (RUs) were recorded. The RU values were collected, and the binding affinity data was calculated by kinetic models (1:1 interaction) within Biacore 8K Evaluation Software. The equilibrium dissociation constant (KD) was calculated to evaluate the ability of ISP I to interact with SelH (Sec44→Cys44).

Co-immunoprecipitation

For co-immunoprecipitation analysis, LN229 wild-type cells, LN229 cells treated with 10 μM of ISP I, and LN229 SELH-KO #2 were harvested, and total protein was prepared from cells using Dynabeads Co-Immunoprecipitation Kit (ThermoFisher) according to the manufacturer’s protocol. Anti-TIF-IA (Abcam, ab112052) antibody

were used for immunoprecipitation. Immunoblotting was then performed using anti-POLI (Cell Signaling, #24799) antibody.

ChIP assay

ChIP assays were performed using a SimpleCHIP Enzymatic Chromatin IP Kit (Magnetic beads) according to manufacturer's instructions (Cell Signaling, #9003). Cross-linked protein-DNA complexes were precipitated by incubating with rabbit anti-POLI (Cell Signaling, #24799) or rabbit IgG (negative control) overnight, and then with magnetic beads for 2 hours. Purified DNA fragments, including rDNA promoter and coding regions, were quantitatively analyzed by real-time PCR with primers against the rDNA promoter and gene body (5'ETS, 5.8S and 28S) following the standard-curve method. Standard curves were created by serial dilution of 2% input chromatin DNA. The values of chromatin DNA precipitated by POLI antibody were normalized to those precipitated by normal rabbit IgG, which is arbitrarily defined as 1. The primer sequences were listed in Supplementary Table 3.

Xenograft and lung metastases mouse models

For intracranial xenografts models, NSG mice were intracranially inoculated with 100,000 LN229-luc cells or 300,000 LN229 WT (or LN229 SELH-KO #2) cells suspended in 2 μL Hank's Balanced Salt Solution (HBSS; Crystalgen, Commack, NY) using a stereotactic device (coordinates, 2 mm anterior and 2 mm lateral from bregma and 2 mm depth from the dura). For mice inoculated with LN229 cells, bioluminescence signals were detected to confirm the survival of tumor cells in mice after one week. Mice were assigned to two groups according to the signal intensity to keep the baseline balanced. ISP I was intraperitoneally injected daily at a dose of 66 mg/kg body weight for 24 days. Mice in control group were injected with the same volume of DMSO dissolved in corn oil. The viability of tumors and total body weight were monitored every 3 days. After 24 days of treatment, mice were euthanized. For mice inoculated with LN229 WT cells or SELH-KO #2 cells, survival end point for animal studies were defined as follows: 1) a loss of more than 15% of body weight, 2) protruded skull, 3) head tile, 4) hunched posture, 5) ataxia, 6) rough hair coat, or 7) impaired mobility.

For subcutaneous xenografts, 8×10^6 786-O cells were injected in the flank of NSG mice, while 5×10^6 IOMM cells were injected in the flank of nude mice. ISP I treatment was initiated after two weeks in the 786-O tumor-bearing mice, while initiated after one

week in the IOMM tumor-tearing mice. Mice were randomly assigned to two groups and treated with normal saline or ISP I (35 mg/kg BW) everyday intraperitoneally. Tumor size (measured with calipers) and total body weight were assessed every 3 days. After 18 days of treatment, mice were euthanized, and tumors were excised and weighed.

For lung metastases studies, 2 $\times 10^5$ B16 mouse melanoma cells or SELH-KO B16 cells were resuspended in 100 μL saline and injected through tail vein of C57BL/6 mice. Mice injected with B16 cells were randomly assigned to two groups and treated daily with normal saline or ISP I (35 mg/kg BW) intraperitoneally. After 12 days of treatment, mice were euthanized, and lungs were harvested to assess lung tumor burden. To establish the mammary carcinoma lung metastases model, BALB/c mice were injected with 3 x 10^5 4T1 cells into the #2 mammary fat pads. Seven days after tumor inoculation, mice were randomized into 2 groups according to the primary tumor size (measured with calipers). ISP I was intraperitoneally injected every day at a dose of 35 mg/kg body weight for 49 days. After 49 days of treatment, mice were euthanized, and lungs were harvested to assess lung tumor burden.

1. Shalem O, Sanjana NE, Hartenian E, Shi X, Scott DA, Mikkelson T, Heckl D, Ebert BL, Root DE, Doench JG *et al*: **Genome-scale CRISPR-Cas9 knockout screening in human cells**. *Science* 2014, **343**(6166):84-87.
2. Luo W, Friedman MS, Shedden K, Hankenson KD, Woolf PJ: **GAGE: generally applicable gene set enrichment for pathway analysis**. *BMC bioinformatics* 2009, **10**:161.

Supplementary Figures

Figure S1

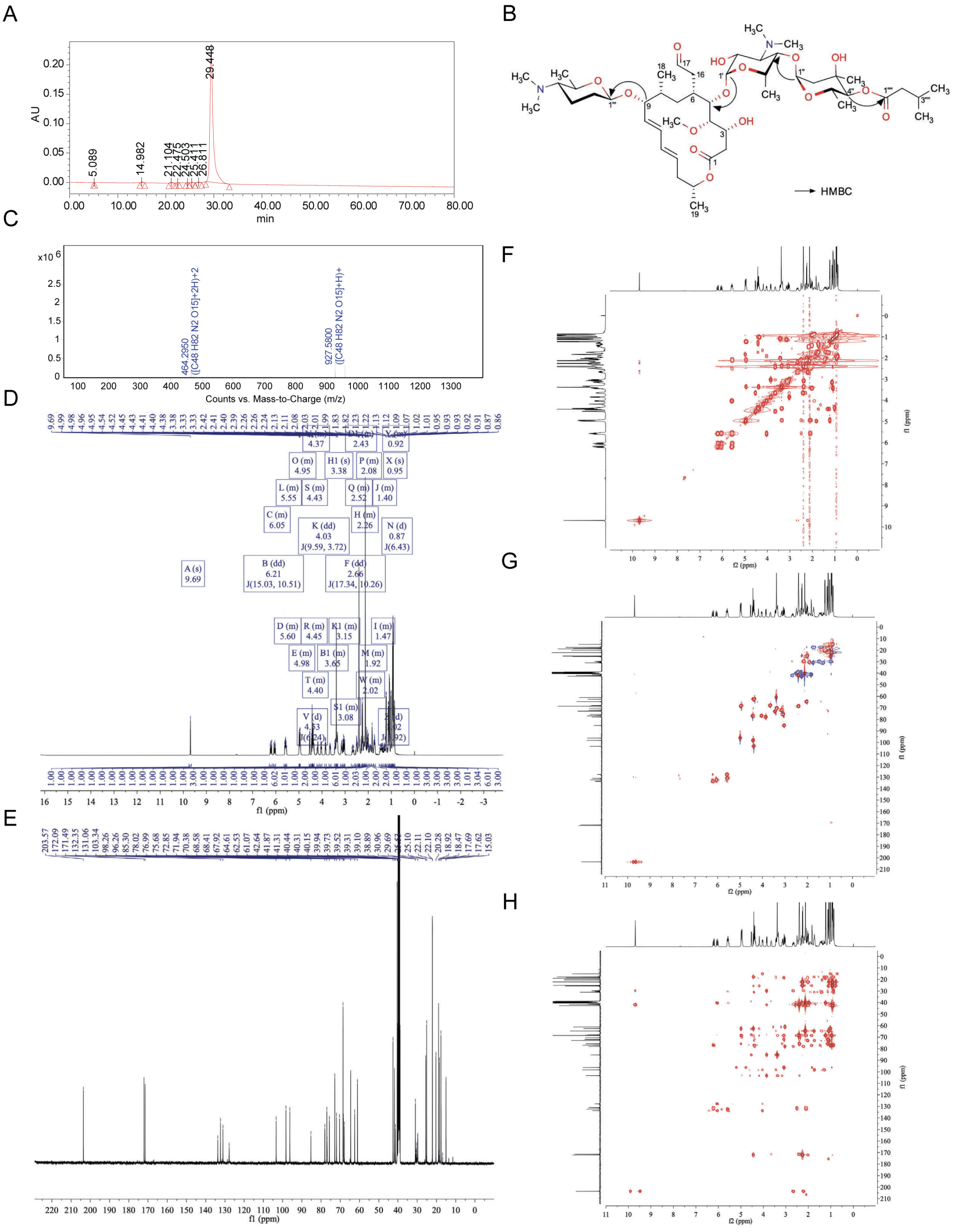

Fig. S1: ISP I structure and purity. **(A)** The HPLC chromatograph of ISP I. **(B)** The structure of ISP I with key HMBC correlations. **(C)** The HRMS spectrum of ISP I. (D) The ^{1}H-NMR spectrum of ISP I. (E) The ^{13}C-NMR spectrum of ISP I. **(F)** The ^{1}H-^{1}H cosy spectrum of ISP I. **(G)** The HSQC spectrum of ISP I. **(H)** The HMBC spectrum of ISP I.

Figure S2

A

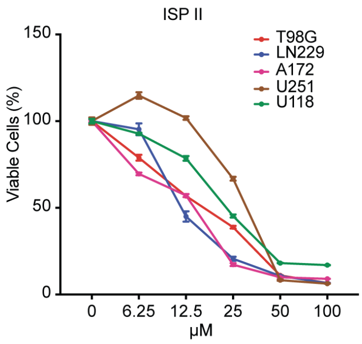

B

Cell line name	ISP II IC50 (μM)
LN229	13.1
A172	12.2
T98G	15.8
U118	23.9
U251	29.2

C

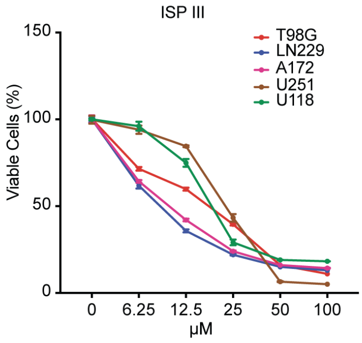

D

Cell line name	ISP III IC50 (μM)
LN229	8.5
A172	9.8
T98G	15.8
U118	19.9
U251	22.5

Fig. S2: Cytotoxicity in glioblastoma cells of ISP II and ISP III. (A and C) Dose-response curve of cell viability was measured by CCK-8 assay in five glioblastoma cell lines (T98G, U118, A172, LN229, and U251) treated with ISP II (A) or ISP III (C) for 48 hours. **(B and D)** IC50 values for the ISP II- (B) and ISP III- (D)-treated glioblastoma cells.

Figure S3

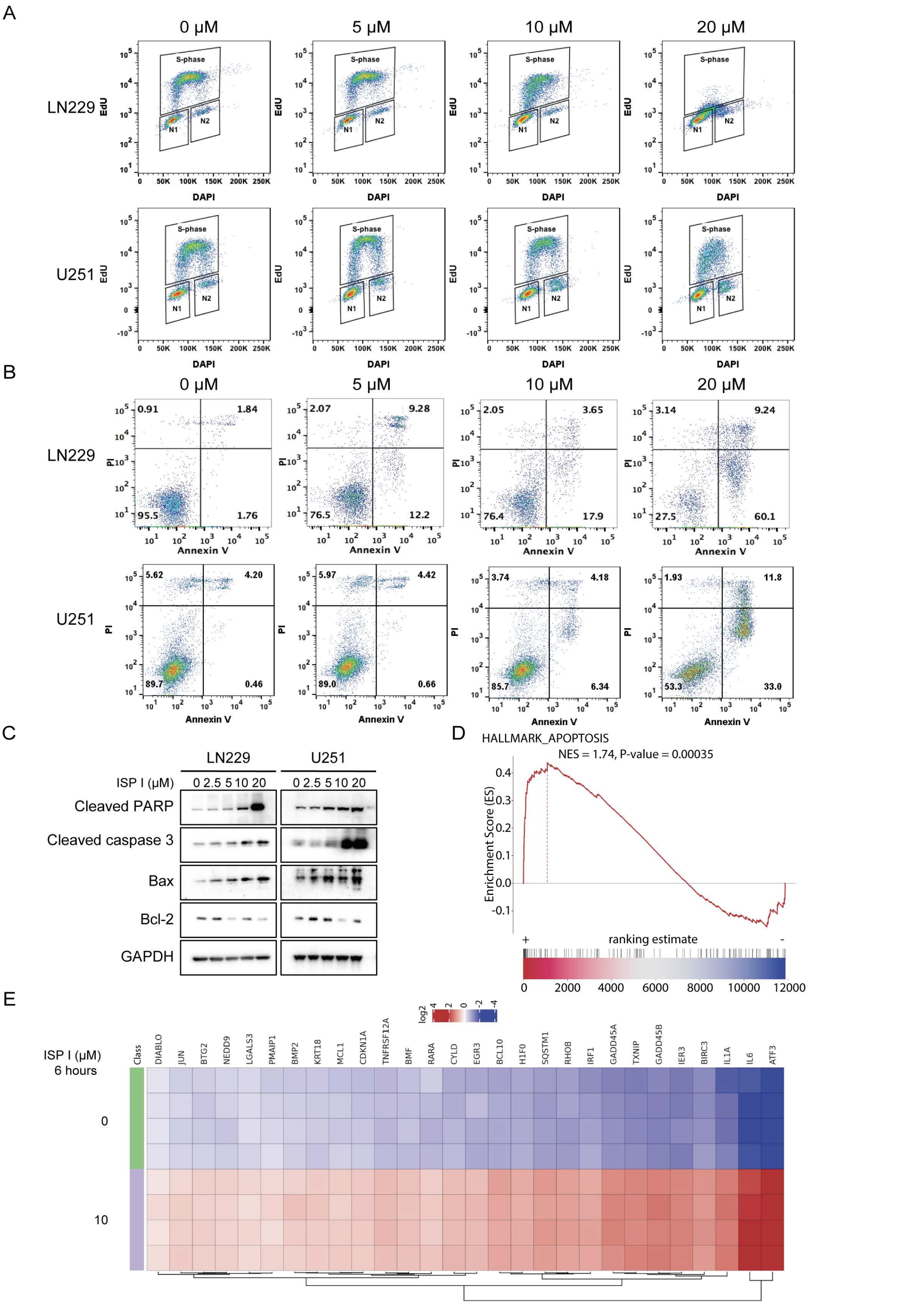

Fig. S3: ISP I leads to cell cycle arrest and apoptosis in glioblastoma cells. (A) Representative flow cytometry scatter plots of cell cycle analysis in ISP I-treated LN229 cells and U251 cells. Cells were treated with ISP I for 6 hours. **(B)** Representative flow cytometry scatter plots of Annexin-V apoptosis analysis in ISP

I-treated LN229 cells and U251 cells. Cells were treated with ISP I for 48 hours. **(C)** Western blots show apoptotic proteins expression of cleaved PARP, cleaved caspase 3, Bax, and Bcl-2 in LN229 cells and U251 cells treated with ISP I at indicated concentrations for 6 hours. **(D and E)** Transcriptomic profiling reveals that ISP I induces an apoptosis pathway. **(D)** Gene Set Enrichment Analysis (GSEA) showing that ISP I-treated cells are highly enriched in genes associated with apoptosis response. NES = 1.74. $P < 0.05$. **(E)** Heatmap of statistically significant differential gene expression as determined by RNA-seq between saline-treated and ISP I-treated cells. LN229 Cells were treated with ISP I at 10 μM or saline for 6 hours. $N = 4$. $P < 0.05$.

Figure S4

A

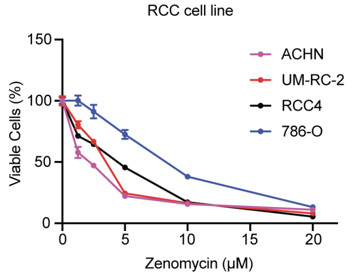

B

Cell line name	IC50 (μM)
ACHN	1.8
UM-RC-2	3.2
RCC4	3.5
786-O	8.0

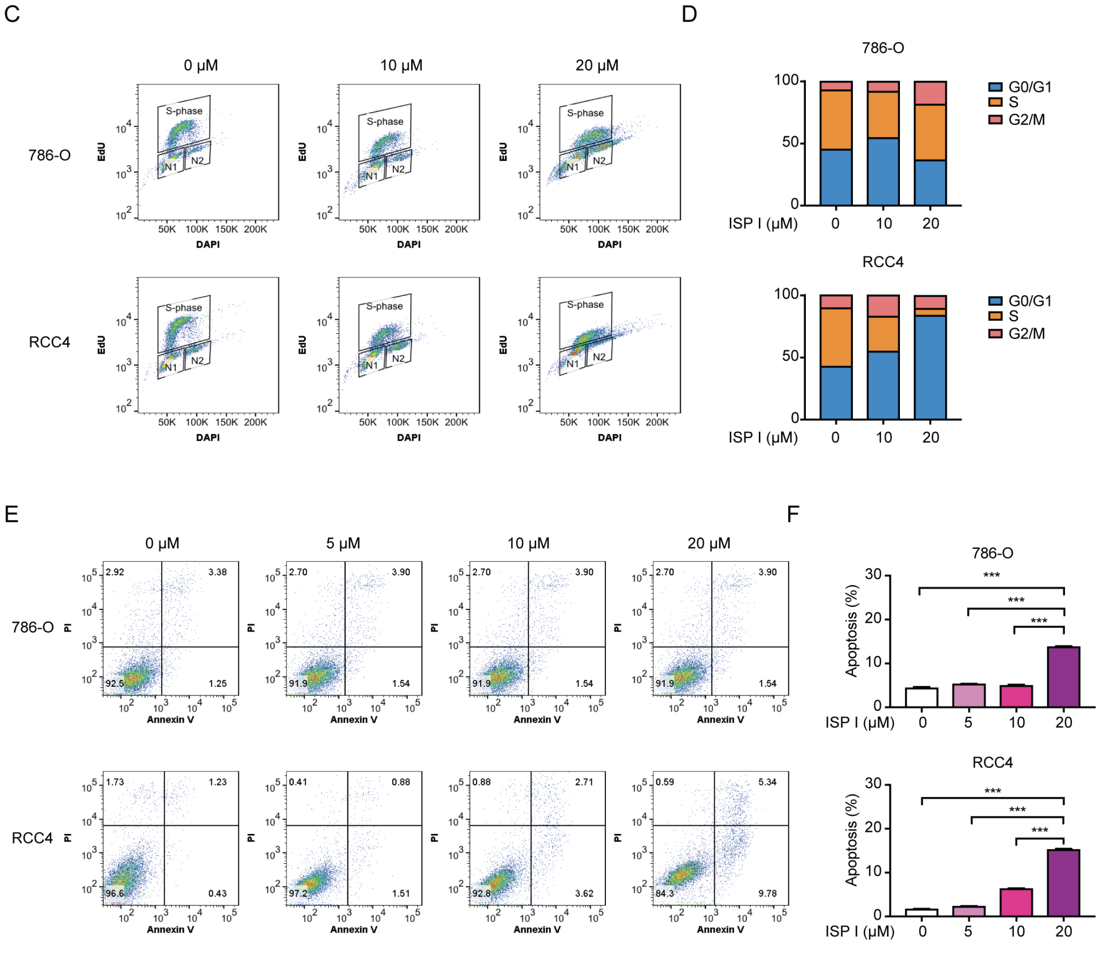

Fig. S4: Cytotoxicity of ISP I in renal cell carcinoma cells. (A) Dose-response curve of cell viability was measured by CCK-8 assay in four renal cell carcinoma (RCC) cell lines (ACHN, UM-RC-2, RCC4 and 786-O) treated with ISP I for 48 hours. **(B)** IC50 values for the ISP I-treated RCC cells. **(C)** Cell cycle analysis of ISP I-treated 786-O and RCC4 cells. **(D)** The overview of cell-cycle progression and arrest in ISP I-treated cells. **(E)** Annexin-V apoptosis analysis of ISP I-treated 786-O and RCC4 cells. **(F)** Summarized results from 4 independent wells are shown. *P* value: $***p < 0.001$.

Figure S5

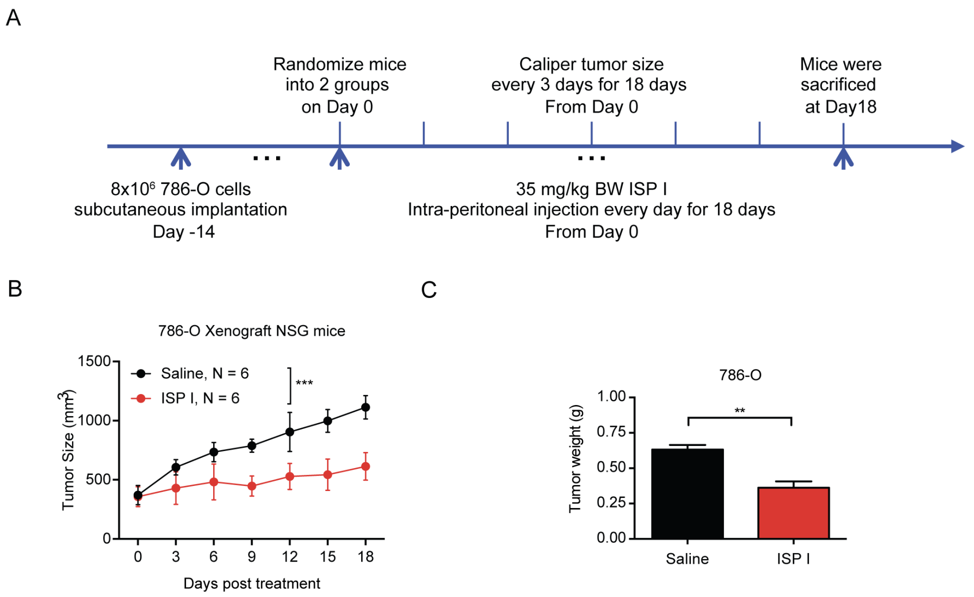

Fig. S5: ISP I suppresses tumor growth in a renal cell carcinoma xenograft mouse model. (A) The schematic outline of the renal cell carcinoma (786-O) xenograft mouse model experiment. NSG mice were randomized into 2 treatment groups: saline (Control) (N = 6) and ISP I (N = 6). **(B)** The tumor growth curve showed reduced 786-O tumor burden in the ISP I treatment arm compared to the saline treatment arm. ***$p < 0.001$ by two-way ANOVA. **(C)** Tumor weight of 786-O xenograft is shown. Tumors were excised and weighed after 18 days of treatment, corresponding to experimental end point. All data are shown as mean ± SEM. P value: **$p < 0.01$.

Figure S6

A

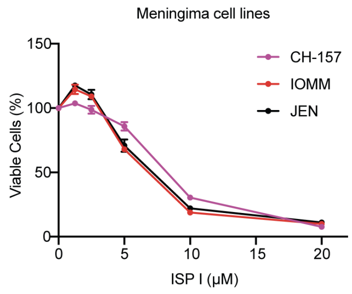

B

Cell line name	IC50 (μM)
IOMM	9.7
JEN	10.3
CH-157	12.1

C

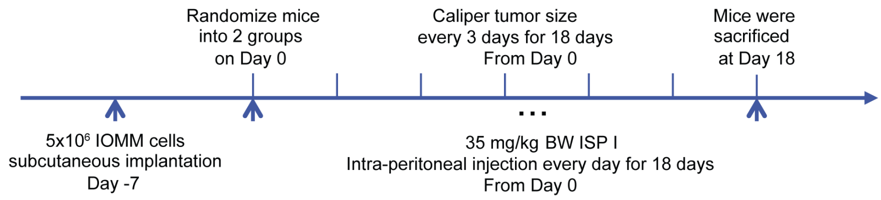

D

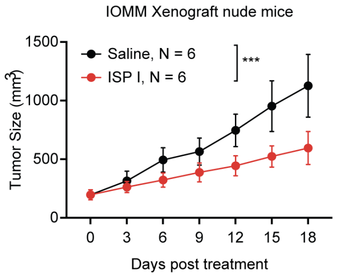

E

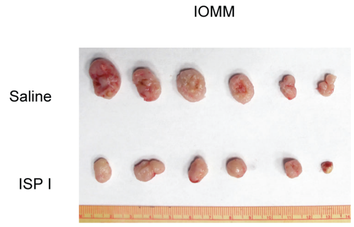

Fig. S6: ISP I suppresses tumor growth in a meningioma xenograft mouse model. (A) Dose-response curve of cell viability was measured by CCK-8 assay in three meningioma cell lines (IOMM, JEN, and CH-157) treated with ISP I for 48 hours. **(B)** IC50 values for the ISP I-treated meningioma cells. **(C)** The schematic outline of the meningioma (IOMM) xenograft mouse model experiment. Nude mice were randomized into 2 treatment groups: saline (Control) (N = 6) and ISP I (N = 6). **(D)** The tumor growth curve showed reduced IOMM tumor burden in the ISP I treatment arm compared to the saline treatment arm. ***p < 0.001 by two-way ANOVA. **(E)** Representative tumors from IOMM xenograft mice. Tumors were excised 18 days after the start of treatment, corresponding to study endpoint. All data are shown as mean ± SEM.

Figure S7

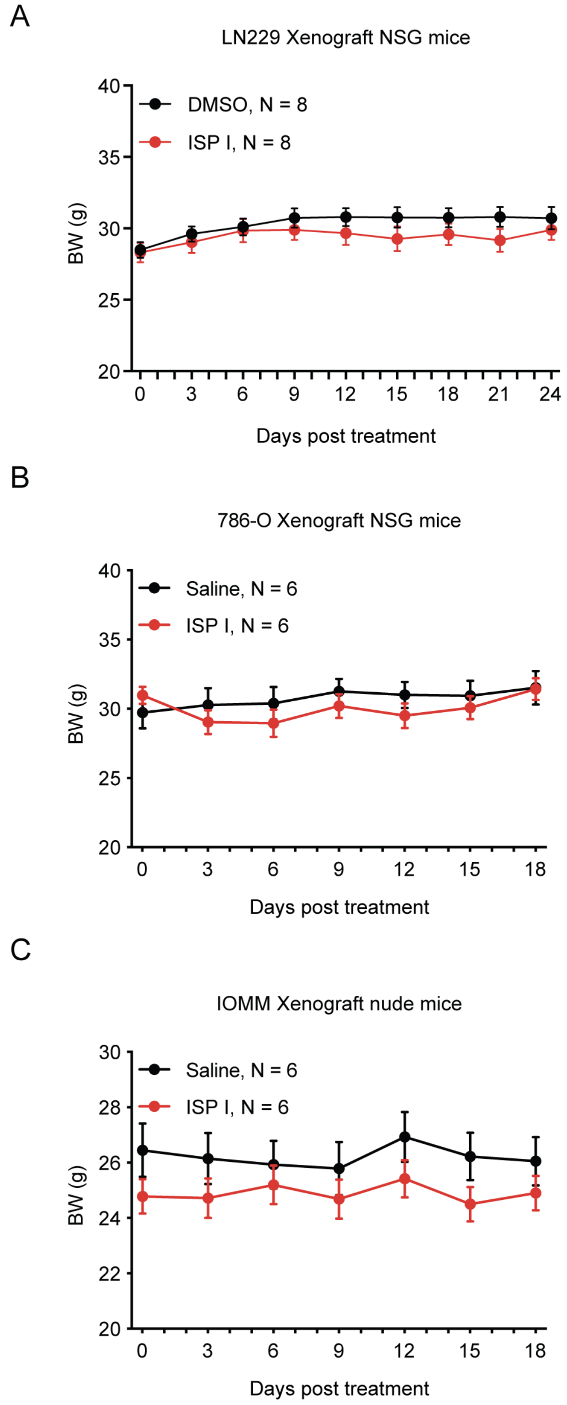

Fig. S7: ISP I treatment is tolerated well. Animals maintained their body weight during ISP I treatment. **(A)** LN229 xenograft NSG mouse model ($N = 8$ per group). **(B)** 786-O xenograft NSG mouse model ($N = 6$ per group). **(C)** IOMM xenograft nude mouse model ($N = 6$ per group). All data are shown as mean ± SEM.

Figure S8

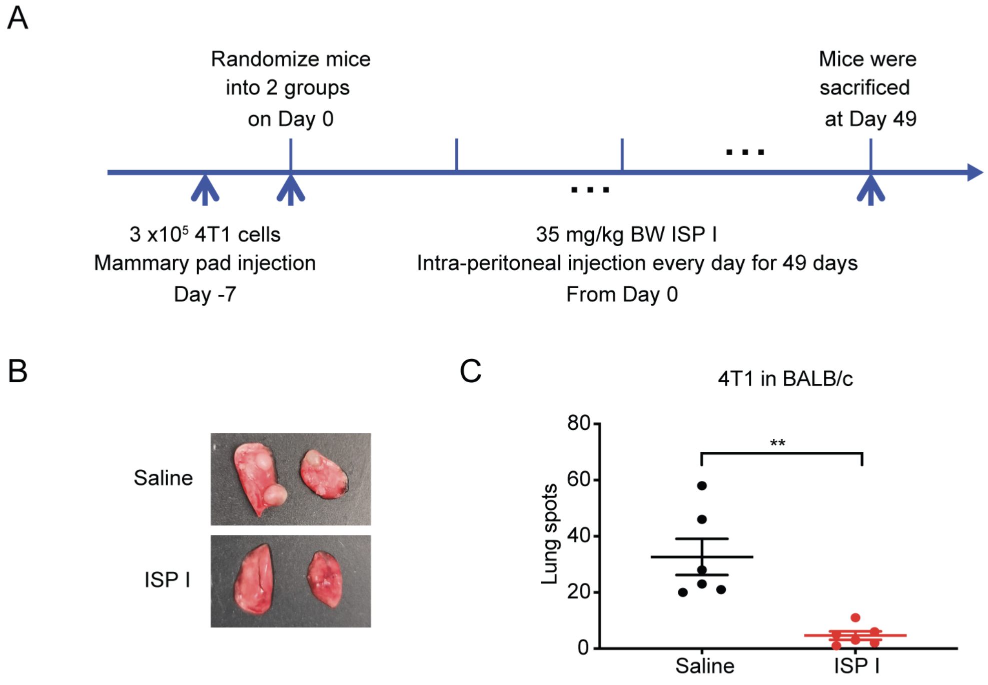

Fig. S8: ISP I reduces metastasis in a breast cancer lung metastasis mouse model. (A) The schematic outline of the breast cancer (4T1) metastasis mouse model experiments. BALB/c mice were randomized into 2 treatment groups: saline (Control) ($N = 6$) and ISP I ($N = 6$). **(B and C)** Representative lungs of mice corresponding to the ISP I and saline treatment arms (B), quantification of lung tumor nodules (C). All data are shown as mean ± SEM. P value: $^{**}p < 0.01$.

Figure S9

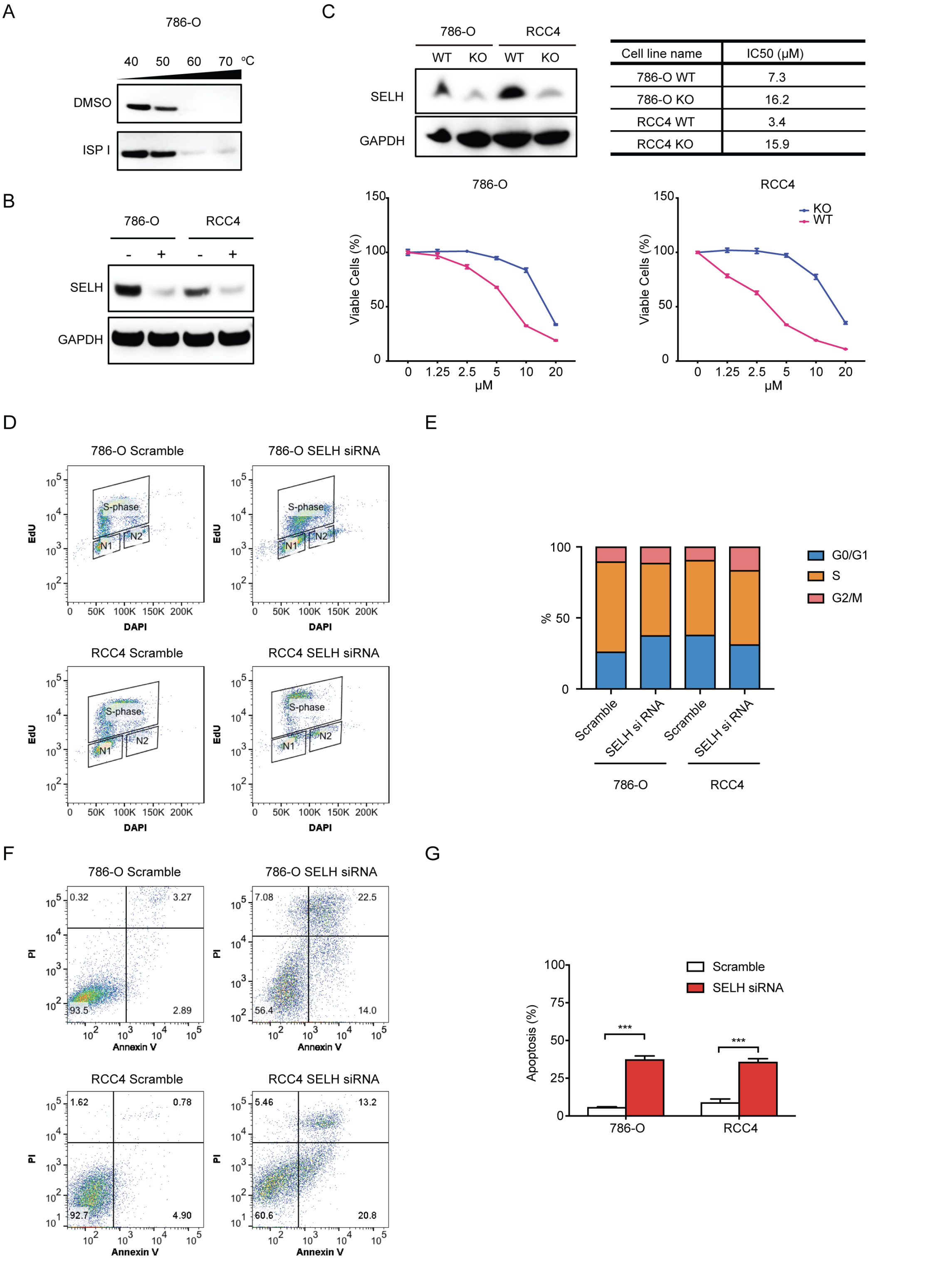

Fig. S9: ISP I targets to SELH in renal cell carcinoma (RCC) cells. (A) The western blots corresponding to thermo-stability assay of ISP I binding to SELH extracted from 786-O cells. DMSO serves as a control. **(B)** Western blots show SELH expression in ISP I-treated RCC cell lines (786-O and RCC4). Cells were treated with 10 μM of ISP I for

24 hours. **(C)** Knock out of SELH in 786-O and RCC4 cells results in resistance to ISP I. Western blots show SELH expression in SELH knockout (KO) 786-O and RCC4 cells. CCK-8 assay was used to measure cell viability. IC50 was calculated and listed. **(D-G)** Deficiency of SELH promotes cell cycle arrest and apoptosis. 786-O and RCC4 cells were transfected with SELH siRNA or scramble siRNA for two days. **(D)** Representative flow cytometry scatter plots of cell cycle analysis in SELH-deficient 786-O and RCC4 cells. **(E)** Summarized results from 4 independent wells of cell cycle analysis are shown. **(F)** Representative flow cytometry scatter plots of Annexin-V apoptosis analysis in SELH-deficient 786-O and RCC4 cells. **(G)** Summarized results from 4 independent wells of Annexin-V apoptosis analysis are shown. Expression of GAPDH serves as an internal control in (B and C). All data are shown as mean ± SEM. *P* value: $^{***}p < 0.001$.

Figure S10

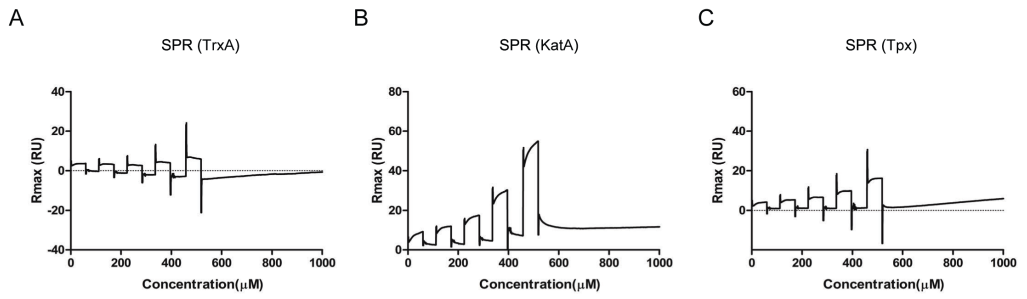

Fig. S10: The specific target of ISP I is SELH. (A-C) Surface plasmon resonance (SPR) assay demonstrating minimal to no interaction between ISP I and the following peroxidases (synthesized in bacteria): Thioredoxin (TrxA) (A), Catalase (KatA) (B), and Thiol peroxidase (TpX) (C).

Figure S11

A

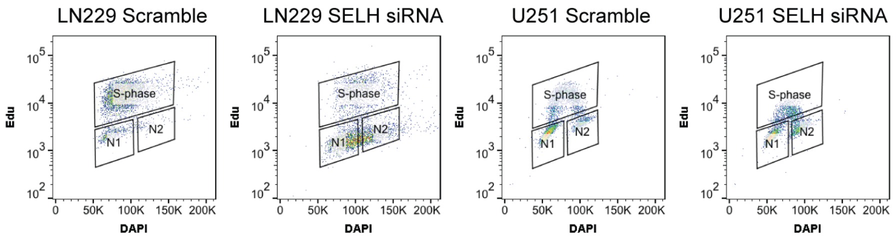

B

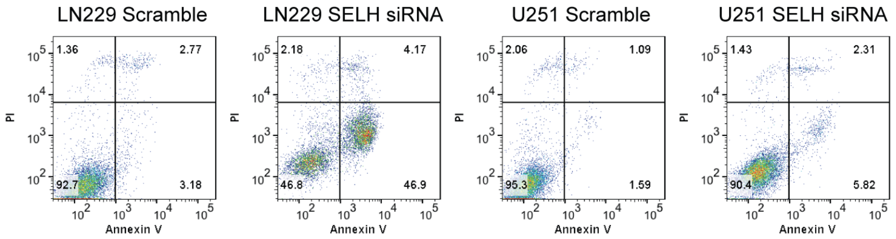

Fig. S11: Deficiency of SELH promotes cell cycle arrest and apoptosis in glioblastoma cells. LN229 and U251 cells were transfected with SELH siRNA or scramble siRNA for two days. **(A)** Representative flow cytometry scatter plots of cell cycle analysis in SELH-deficient LN229 and U251 cells. **(B)** Representative flow cytometry scatter plots of Annexin-V apoptosis analysis in SELH-deficient LN229 and U251 cells.

Figure S12

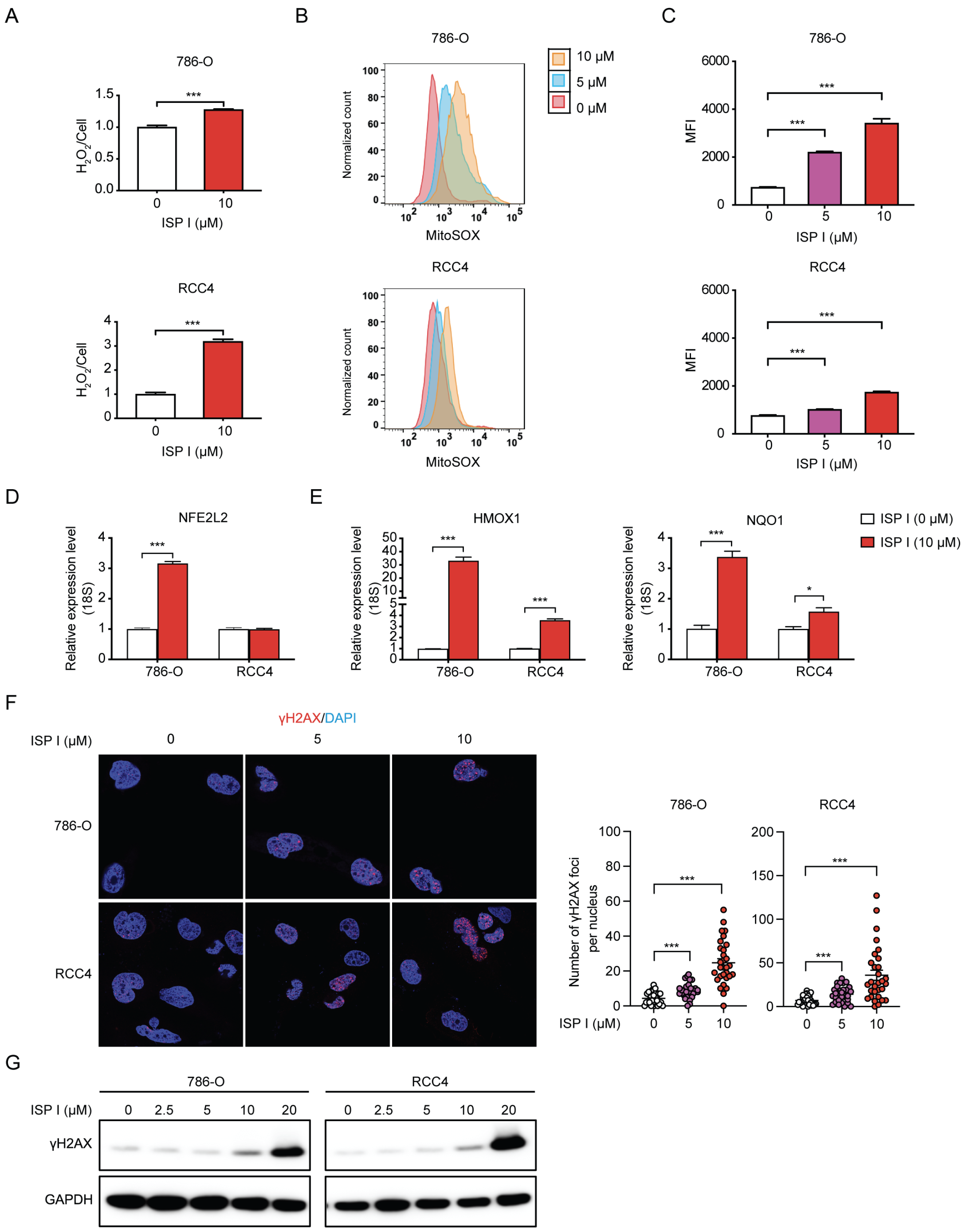

Fig. S12: ISP I triggers ROS accumulation and DNA damage in RCC cells. (A) Quantification of intracellular reactive oxygen species (ROS) levels by ROS-Glo H_2O_2 assay. 786-O and RCC4 cells were treated with ISP I at 10 μM for 24 hours. All groups were normalized to saline-treated groups. **(B)** Flow cytometry analysis of ROS level using MitoSOX staining in ISP I-treated 786-O and RCC4 cells. Cells were pre-treated with 5 μM or 10 μM ISP I for 24 hours. **(C)** Summarized ROS levels by mean fluorescence intensity (MFI) of MitoSOX positive cells from 3 independent wells are

shown. **(D)** Real-time RT-PCR analysis shows *NFE2L2* mRNA in RCC cell lines (786-O and RCC4) treated with 10 μM of ISP I for 24 hours. 18S expression serves as an internal control. **(E)** Real-time RT-PCR results show the mRNA expression of NFE2L2 downstream gene-*HMOX1* and *NQO1* with 18S expression serving as an internal control. **(F and G)** immunofluorescence staining assay (F) and western blots (G) show γH2AX expression in 786-O and RCC4 cells treated with ISP I at indicated concentrations for 6 hours. The number of γH2AX foci in cell nucleus was identified and quantified. Expression of GAPDH serves as an internal control. All data are shown as mean ± SEM. *P* value: $*p < 0.05$; $***p < 0.001$.

Figure S13

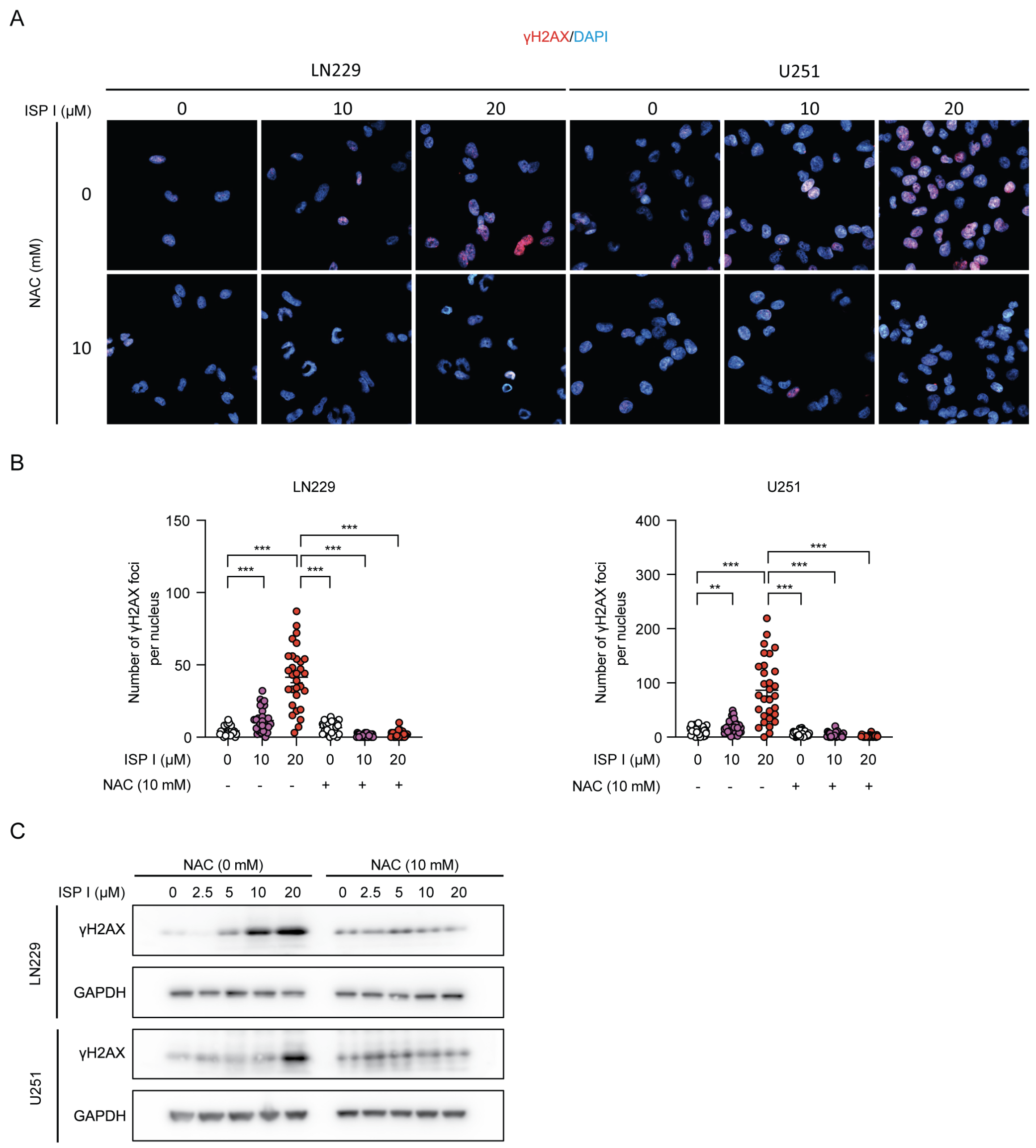

Fig. S13: N-acetylcysteine rescues ISP I-induced DNA damage. **(A-C)** Immunofluorescence staining assay (A and B) and western blots (C) show γH2AX expression in LN229 and U251 cells treated with ISP I and N-acetylcysteine (NAC) at indicated concentrations for 6 hours. NAC was added at 10 mM as a ROS scavenger. The number of γH2AX foci in cell nucleus was identified and quantified (B). Expression of GAPDH serves as an internal control. All data are shown as mean ± SEM. *P* value: $^{**}p < 0.01$; $^{***}p < 0.001$.

Figure S14

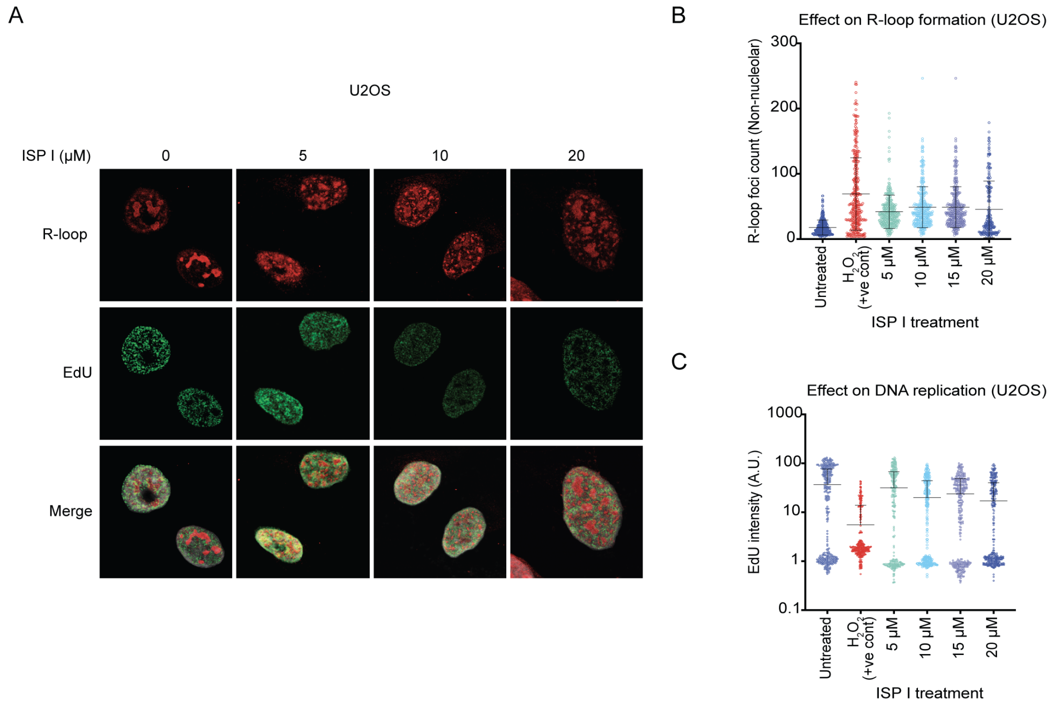

Fig. S14: ISP I triggers R-loop formation. (A-C) Immunofluorescence staining assay shows an increase of R-loop formations and a simultaneous decrease in EdU incorporation in U2OS cells. U2OS cells stably expressed a V5-tagged catalytically dead RNaseH1 for recognition of R-loops, and then treated with ISP I at indicated concentrations for 6 hours. U2OS cells were co-stained with antibodies recognizing R-loop (V5, red) and DNA replication (EdU, green). Representative immunofluorescence staining images are shown in (A). Quantitative comparations of R-loop formations (B) and DNA replication (C) are shown. All data are shown as mean ± SEM. *P* value: **p* < 0.05; ***p* < 0.01; ****p* < 0.001.

Figure S15

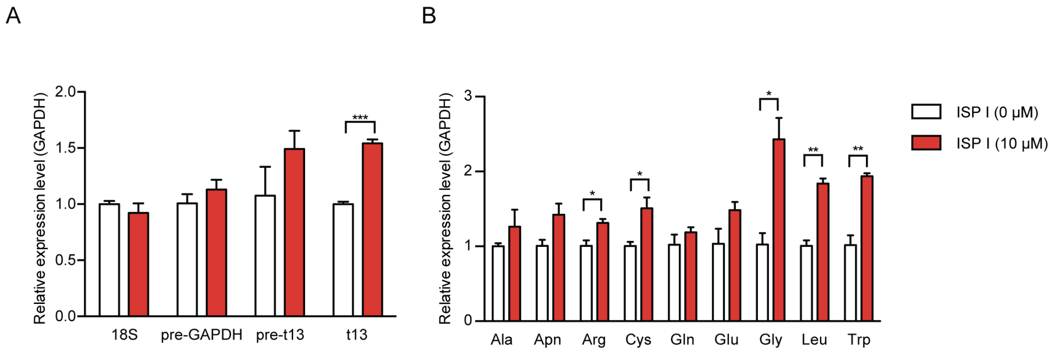

Fig. S15: ISP I alters nucleolar function. (A) Real-time RT-PCR analysis shows RNA levels of 18S RNA (rRNA cleavage), pre-GAPDH (POL II transcript) expression, and pre-tRNA 13 (POL III transcript) in ISP I-treated cells. LN229 cells were treated with ISP I at 10 μM for 8 hours. **(B)** Real-time RT-PCR analysis shows different RNA levels of the following mature tRNA expression in ISP I-treated LN229 cells: Arg, Cys, Gly, Leu and Trp. *GAPDH* served as an internal control. All data are shown as mean ± SEM. *P* value: *$p < 0.05$; **$p < 0.01$; ***$p < 0.001$.

Table S1. ^{1}H and ^{13}C-NMR data (400/100 MHz) of ISP I in DMSO-*d_6* (δ in ppm, *J* in Hz.)

No.	δ_H (*J*)	δC, Type	HMBC
1		171.49, C	
2	2.32, m	38.89, CH_2	
	2.43, m		C1
3	3.65, m	67.92, CH	
4	3.03, m	85.30, CH	4-OCH_3, C5
4-OCH_3	3.38, s	61.07, CH_3	C4
5	3.85, d (8.61)	78.02, CH	C4, C6, C7
6	2.18, m	29.69, CH	C16
7	0.98, m	29.78, CH_2	
	1.47, m		C8, C9
8	1.92, m	30.38, CH	C9
9	4.03, dd (9.59, 3.72)	76.87, CH	C11, C18, C1'''
10	5.60, m	127.77, CH	C8
11	6.21, dd (15.03, 10.51)	133.73, CH	C10, C12, C13, C14
12	6.05, m	132.35, CH	C11
13	5.55, m	131.06, CH	
14	2.08, m	40.31, CH_2	C12, C13, C15, C19
	2.52, m		C12, C13, C15
15	4.95, m	68.41, CH	
16	2.23, m	41.87, CH_2	C5, C6, C7, C17
	2.66, dd (17.34, 10.26)		C5, C6, C7, C17
17	9.69, s	203.57, CH	C6, C16
18	0.87, d (6.43)	15.03, CH_3	C6, C7, C8, C9
19	1.23, d (6.17)	20.28, CH_3	C14, C15
1'	4.40, m	103.34, CH	C5, C5'

2'	3.34, m	70.38, CH	C1'
3'	2.39, m	68.58, CH	C1', C2', C4', 3'-N(CH_3)$_2$
4'	3.08, m	75.68, CH	C3', C5', C6'
5'	3.15, m	71.94, CH	C1', C4'
6'	1.12, d (5.95)	18.47, CH_3	C4', C5'
3'-N(CH_3)$_2$	2.40, s	41.87, CH_3	C3'
	2.40, s	41.87, CH_3	C3'
1''	4.98, m	96.26, CH	C4', C3''
2''	1.83, m	41.31, CH_2	C1'', C3'', C4'', C5''
3''		68.58, C	
4''	4.45, m	76.99, CH	3''-CH_3, C1''''
5''	4.37, m	62.53, CH	C4'', C6''
6''	1.02, d (5.92)	17.69, CH_3	C5''
3''-CH_3	0.95, s	25.53, CH_3	C1'', C2'', C3'', C4''
1'''	4.43, m	98.26, CH	C2'''
2'''	1.30, m	30.96, CH_2	C1''', C3'''
	1.73, m		C1''', C3'''
3'''	1.40, m	17.62, CH_2	C2''', C4'''
	1.73, m		C2''', C4''', C5'''
4'''	2.02, m	64.61, CH	C5''', C6''', 4'''-N(CH_3)$_2$
5'''	3.44, m	72.85, CH	C1'''
6'''	1.08, d (6.03)	18.92, CH_3	C3''', C4''', C5'''
4'''-N(CH_3)$_2$	2.13, s	40.44, CH_3	C4'''
	2.13, s	40.44, CH_3	C4'''
1''''		172.09, C	
2''''	2.26, m	42.64, CH_2	C1'''', C3'''', C4'''', C5''''
3''''	2.02, m	25.10, CH	C1'''', C2'''', C4'''', C5''''
4''''	0.92, m	22.10, CH_3	C2'''', C3''''

5''''	0.92, m	22.11, CH_3	C2'''', C3''''

Table S2. DARTS-MS

Gene Symbol	Accession	Description	Abundance Ratio: (ISP I) / (DMSO)	Abundance Ratio P-Value: (ISP I) / (DMS	Abundance Ratio: (DMSO-C*) / (DMSO)	Abundance Ratio P-Value: (DMSO-C) / (DMSO)	Abundance Ratio: (ISP I-C) /	Abundance Ratio P-Value: (ISP I-C) / (ISP I)
RPL37A	P61513	60S ribosomal protein L37a	3.304	2.62373E-08	11.516	0.002380672	5.818	0.027124604
SELH	Q8IZQ5	Selenoprotein H	3.175	1.39638E-06	13.934	0.003301563	2.886	0.249292894
API5	Q9BZZ5	Apoptosis inhibitor 5	2.758	7.79136E-07	20.128	6.34671E-05	4.086	0.083205214
HYPK	Q9NX55	Huntingtin-interacting protein K	2.75	1.05837E-07	11.833	0.000503263	3.157	0.135364309
ACTG1	P63261	Actin, cytoplasmic 2	2.735	4.33108E-06	4.068	0.188140852	3.325	0.156350523
AGL	P35573	Glycogen debranching enzyme	2.642	4.04876E-05	3.325	0.263637677	1.375	0.872230624
PPP2R5E	Q16537	Serine/threonine-protein phosphatase 2A 56 kDa regulatory subunit epsilon isoform	2.533	0.000171708	7.525	0.035525202	1.689	0.657846161
TPP1	O14773	Tripeptidyl-peptidase 1	2.452	8.02442E-05	18.385	0.000167037	5.998	0.022950709
CLEC3B	P05452	Tetranectin	2.374	6.06991E-09	3.177	0.202143917	1.707	0.628898357
SPARC	P09486	SPARC	2.293	2.70608E-07	22.092	3.1841E-07	10.058	0.00021103
COPS3	Q9UNS2	COP9 signalosome complex subunit 3	2.227	2.34594E-06	3.632	0.164898494	1.378	0.958734522
GSTK1	Q9Y2Q3	Glutathione S-transferase kappa 1	2.129	1.73691E-08	4.01	0.071924976	1.414	0.839544547
FAM82B; RMDN1	Q96DB5	Regulator of microtubule dynamics protein 1	2.124	0.001485071	1.764	0.690885361	1.531	0.760761353
HTRA1	Q92743	Serine protease HTRA1	1.99	0.002470256	12.585	0.001570747	2.979	0.205898687
NDRG3	Q9UGV2	Protein NDRG3	1.985	0.003325075	9.913	0.004738697	4.033	0.100077276
RAB10	P61026	Ras-related protein Rab-10	1.967	2.13885E-08	4.261	0.049429299	1.8	0.540808055
DPP9	Q86TI2	Dipeptidyl peptidase 9	1.922	0.012439695	3.253	0.271338449	1.679	0.675127785
ACSS1	Q9NUB1	Acetyl-coenzyme A synthetase 2-like, mitochondrial	1.898	8.58564E-05	2.34	0.433363696	1.062	0.746710037
ITPK1	Q13572	Inositol-tetrakisphosphate 1-kinase	1.88	0.000384972	2.82	0.31584353	2.017	0.457012655
STK25	O00506	Serine/threonine-protein kinase 25	1.876	0.000420495	5.634	0.037580471	1.847	0.560153528
PARVA	Q9NVD7	Alpha-parvin	1.825	0.001223703	10.867	0.001072589	1.446	0.881686491
COA4	Q9NYJ1	Cytochrome c oxidase assembly factor 4 homolog, mitochondrial	1.821	0.015507737	10.614	0.009174414	3.42	0.173325724
ARF4	P18085	ADP-ribosylation factor 4	1.82	0.002286281	16.673	1.50909E-05	10.032	0.000326867
GCLM	P48507	Glutamate--cysteine ligase regulatory subunit	1.815	0.00886243	5.899	0.037600883	7.473	0.006464194
TXNDC9	O14530	Thioredoxin domain-containing protein 9	1.77	0.02728616	12.645	0.004694132	3.366	0.173709616
RRM2B	Q7LG56	Ribonucleoside-diphosphate reductase subunit M2 B	1.754	0.014708179	2.804	0.373044033	1.725	0.655094288
TBCA	O75347	Tubulin-specific chaperone A	1.752	2.2821E-10	9.465	1.02593E-05	3.879	0.019960389
CLTA	P09496	Clathrin light chain A	1.75	3.90454E-08	11.085	4.28548E-06	3.144	0.062496275
RHOA	P61586	Transforming protein RhoA	1.747	2.79968E-06	4.953	0.017362243	1.574	0.702580914
ACTC1	P68032	Actin, alpha cardiac muscle 1	1.736	4.45857E-10	17.699	5.63215E-09	11.39	2.48855E-06
TUBA4A	P68366	Tubulin alpha-4A chain	1.734	3.30862E-05	13.53	3.51501E-06	8.794	8.29248E-05
TUBB4B	P68371	Tubulin beta-4B chain	1.726	0.000581244	27.662	3.85509E-11	15.189	5.78544E-07
CALU	O43852	Calumenin	1.725	7.21693E-10	20.433	7.63985E-10	11.162	3.09571E-06
RRM1	P23921	Ribonucleoside-diphosphate reductase large subunit	1.704	0.000545408	3.526	0.112887733	2.005	0.452349595
GLMN	Q92990	Glomulin	1.704	0.041939362	3.011	0.31286529	1.037	0.824601232
RAB13	P51153	Ras-related protein Rab-13	1.697	0.028374946	4.742	0.115130682	2.569	0.301298806
COX17	Q14061	Cytochrome c oxidase copper chaperone	1.687	0.048822174	32.032	7.58548E-06	7.082	0.011203468
RPL36A	P83881	60S ribosomal protein L36a	1.681	2.73389E-06	4.964	0.014066641	2.09	0.386466076
RUVBL1	Q9Y265	RuvB-like 1	1.68	9.74403E-07	4.755	0.017680612	2.164	0.368358207
UGDH	O60701	UDP-glucose 6-dehydrogenase	1.671	5.67272E-05	8.893	0.000589162	5	0.016375855
PHGDH	O43175	D-3-phosphoglycerate dehydrogenase	1.663	1.24861E-08	12.875	3.26384E-07	5.235	0.002788759
AK1	P00568	Adenylate kinase isoenzyme 1	1.662	2.33218E-05	10.984	6.0119E-05	5.897	0.004285486
PPP2R5D	Q14738	Serine/threonine-protein phosphatase 2A 56 kDa regulatory subunit delta isoform	1.66	0.004117143	6.226	0.013017265	2.942	0.158007543
HNRNPA0	Q13151	Heterogeneous nuclear ribonucleoprotein A0	1.649	0.003399136	3.232	0.146649217	1.463	0.863606267
PRKAR1A	P10644	cAMP-dependent protein kinase type I-alpha regulatory subunit	1.641	7.88448E-08	18.644	2.76417E-09	7.639	0.000130293
ARF3	P61204	ADP-ribosylation factor 3	1.62	3.59044E-05	12.629	7.66474E-07	5.427	0.005434881
PBK	Q96KB5	Lymphokine-activated killer T-cell-originated protein kinase	1.608	0.000966947	5.302	0.020093507	3.529	0.079895277
CLASP1	Q7Z460	CLIP-associating protein 1	1.608	0.021592199	2.25	0.525477387	1.197	0.936853072
KLC1	Q07866	Kinesin light chain 1	1.605	0.013887908	4.364	0.079150834	1.547	0.795097308
RIC8A	Q9NPQ8	Synembryn-A	1.599	0.049493427	5.399	0.080848294	2.627	0.287062027
SRP19	P09132	Signal recognition particle 19 kDa protein	1.586	0.034286933	4.023	0.117052904	1.083	0.775385902
RPL35	P42766	60S ribosomal protein L35	1.578	0.027872328	66.981	8.4821E-14	17.474	4.06369E-06
TUBB	P07437	Tubulin beta chain	1.575	3.52162E-07	16.198	1.83403E-08	8.178	6.99011E-05
EIF4A3	P38919	Eukaryotic initiation factor 4A-III	1.572	1.18702E-05	3.099	0.129911923	1.198	0.866594481
PLCH1	Q4KWH8	1-phosphatidylinositol 4,5-bisphosphate phosphodiesterase eta-1	1.572	0.010272921	11.219	8.20989E-05	8.255	0.001050821
EEF1B2	P24534	Elongation factor 1-beta	1.569	4.4503E-07	4.545	0.005918857	1.992	0.393221312
PDCD5	O14737	Programmed cell death protein 5	1.562	0.000991442	4.828	0.01984733	2.44	0.252617609
FAM50A	Q14320	Protein FAM50A	1.559	0.024335935	2.849	0.297031615	2.861	0.219773497
FARSA	Q9Y285	Phenylalanine--tRNA ligase alpha subunit	1.547	0.00489511	5.191	0.017611292	3.428	0.098204316
ARF5	P84085	ADP-ribosylation factor 5	1.542	0.044773573	18.292	9.42807E-06	12.091	0.000115368
CLTB	P09497	Clathrin light chain B	1.541	0.030636166	10.062	0.000616368	4.395	0.033839196
LZIC	Q8WZA0	Protein LZIC	1.54	0.016371485	3.789	0.12882647	1.554	0.806504641
RPL23A	P62750	60S ribosomal protein L23a	1.536	0.00043596	21.81	2.98078E-10	5.032	0.00862887
ACTB	P60709	Actin, cytoplasmic 1	1.535	0.036396085	28.984	1.07499E-08	18.464	2.71848E-06
SPIRE2	Q8WWL2	Protein spire homolog 2	1.533	0.000430755	12.168	2.39239E-06	6.203	0.003301607
CBR3	O75828	Carbonyl reductase [NADPH] 3	1.525	0.02464157	8.937	0.0024129	5.33	0.015113544
PEA15	Q15121	Astrocytic phosphoprotein PEA-15	1.518	1.4521E-05	2.769	0.109767759	1.609	0.733068924
HAT1	O14929	Histone acetyltransferase type B catalytic subunit	1.518	0.000188986	4.196	0.032231367	3.37	0.094875267
RPL27A	P46776	60S ribosomal protein L27a	1.516	6.9812E-05	3.75	0.040239836	2.024	0.441822648
TOMM34	Q15785	Mitochondrial import receptor subunit TOM34	1.515	0.027334214	5.685	0.025411035	2.725	0.237376484
RPL34	P49207	60S ribosomal protein L34	1.512	1.43095E-05	3.726	0.034512978	1.405	0.957271437
ACOT7	O00154	Cytosolic acyl coenzyme A thioester hydrolase	1.505	0.000107247	5.951	0.003931692	3.63	0.050753765
RPL23	P62829	60S ribosomal protein L23	1.501	0.000247323	10.434	7.84619E-05	2.81	0.15287438
RPS7	P62081	40S ribosomal protein S7	1.492	8.10346E-06	4.957	0.003193587	2.178	0.293385149
RPS23	P62266	40S ribosomal protein S23	1.489	6.56822E-05	9.568	9.16298E-06	5.327	0.002455997
SCP2	P22307	Non-specific lipid-transfer protein	1.48	0.000700096	4.555	0.030927743	3.187	0.094307273
VCP	P55072	Transitional endoplasmic reticulum ATPase	1.467	1.98738E-05	3.063	0.062900333	1.977	0.402368167
IPO4	Q8TEX9	Importin-4	1.461	0.003613521	2.04	0.481116802	1.266	0.98863028
MARS	P56192	Methionine--tRNA ligase, cytoplasmic	1.459	0.014497332	5.671	0.011096268	3.689	0.066268238
SRP68	Q9UHB9	Signal recognition particle subunit SRP68	1.454	0.006511272	1.504	0.857040445	1.095	0.783828035
PTMA	P06454	Prothymosin alpha	1.452	3.41669E-05	4.258	0.009190622	2.66	0.135423293

C*: lysate without Pronase digestion as a control

Table S3. Primers for real-time RT-PCR and CHIP-qPCR

**Name**	**Sequence**
18S-F	CGCCGCTAGAGGTGAAATTCT
18S-R	CGAACCTCCGACTTTCGTTCT
NFE2L2-F	CAACATCCAGCTCTTTGAGG
NFE2L2-R	TGGGCAACCTGGGAGTAG
HMOX1-F	CAACATCCAGCTCTTTGAGG
HMOX1-R	GGCAGAATCTTGCACTTTG
NQO1-F	AGCCCAGATATTGTGGCCG
NQO1-R	CCTTTCAGAATGGCTGGCAC
pre-rRNA-F	TGTCAGGCGTTCTCGTCTC
pre-rRNA-R	AGCACGACGTCACCACATC
Pre-GAPDH-F	GAGCTGGGGAATGGGACT
Pre-GAPDH-R	TGATGGCATGGACTGTGG
GAPDH-F	GAGTCAACGGATTTGGTCGT
GAPDH-R	TTGATTTTGGAGGGATCTCG
pre-t13-F	CACCCTGATAGAGCCATCAC
pre-t13-R	CTCTGCATGTACTGCTGTATAAGTACC
t13-F	GTGGCGCAATCGGTTAGC
t13-R	TGCTCCAGGTGAGGCTCG
Ala-F	GGGGAATTAGCTCAAGCGGTAG
Ala-R	TAGAGAATGGGGGCGTCGATC
Apn-F	GTCTCTGTGGCGCCATCGGTTAG
Apn-R	CATCTCTGGGTGGGCTTGAAC
Arg-F	GTCTCTGTGGCGCAATGGAC
Arg-R	CATCTCTGCCGGGACTCGAAC
Cys-F	GGGGGTAGGGCTCAGGGATAG

Cys-R	AGGGGGCACCTAGATTCGAAC
Gln-F	GGCAGTATGGTAGAGTGGTTAAG
Gln-R	TGACAGAACCAGCATTCAAATTC
Glu-F	TCCTTGATGTCTAGTGGTTAG
Glu-R	TTCCCTGACTGAGAAATGAAC
Gly-F	GCATTGGTGGTTCAGTGGTAG
Gly-R	TGCATTGGCCAGGAATCGAAG
Leu-F	GGCAGTGGAGTTTAGTGGTTAAG
Leu-R	TGCCAGGGCTAGGGTTTGAAC
Trp-F	GGGGAATTAGCTCAAGCGGTAG
Trp-R	TAGAGAATGGGGGCGTCGATC
rDNA promoter-CHIP-F	GCTGCGATGGTGGCGTTTTTGGGG
rDNA promoter-CHIP-R	ATATAACCCGGCGGCCCAAAATTGCC
5ETS-CHIP-F	CGTGCCTGAGGTTTCTCC
5ETS-CHIP-R	CCACCAACGGACGTGAAG
5.8S-CHIP-F	GCAGGACACATTGATCATCGACAC
5.8S-CHIP-R	GCGCGGCGGCAAGAGGAG
28S-CHIP-F	GGAGGAAAAGAAACTAACCAGGAT
28S-CHIP-R	GCCTCGATCAGAAGGACTTG
